# Mov10 suppresses retroelements and regulates neuronal development and function in the developing brain

**DOI:** 10.1186/s12915-017-0387-1

**Published:** 2017-06-29

**Authors:** Geena Skariah, Joseph Seimetz, Miles Norsworthy, Monica C. Lannom, Phillip J. Kenny, Mohamed Elrakhawy, Craig Forsthoefel, Jenny Drnevich, Auinash Kalsotra, Stephanie Ceman

**Affiliations:** 1 Neuroscience Program, University of Illinois-Urbana Champaign, Urbana, IL 61801 USA; 2 Cell and Developmental Biology, University of Illinois-Urbana Champaign, Urbana, IL 61801 USA; 3 Biochemistry, University of Illinois-Urbana Champaign, Urbana, IL 61801 USA; 4 College of Medicine, University of Illinois-Urbana Champaign, Urbana, IL 61801 USA; 5 High-Performance Biological Computing, Roy J. Carver Biotechnology Center, University of Illinois-Urbana Champaign, Urbana, IL 61801, USA

**Keywords:** RNA helicase, RISC, Brain, Neurite outgrowth, Embryonic development, Mov10, Retrotransposons, L1, Neurogenesis

## Abstract

**Background:**

Moloney leukemia virus 10 (Mov10) is an RNA helicase that mediates access of the RNA-induced silencing complex to messenger RNAs (mRNAs). Until now, its role as an RNA helicase and as a regulator of retrotransposons has been characterized exclusively in cell lines. We investigated the role of Mov10 in the mouse brain by examining its expression over development and attempting to create a Mov10 knockout mouse. Loss of both Mov10 copies led to early embryonic lethality.

**Results:**

Mov10 was significantly elevated in postnatal murine brain, where it bound retroelement RNAs and mRNAs. Mov10 suppressed retroelements in the nucleus by directly inhibiting complementary DNA synthesis, while cytosolic Mov10 regulated cytoskeletal mRNAs to influence neurite outgrowth. We verified this important function by observing reduced dendritic arborization in hippocampal neurons from the Mov10 heterozygote mouse and shortened neurites in the Mov10 knockout Neuro2A cells. Knockdown of Fmrp also resulted in shortened neurites. Mov10, Fmrp, and Ago2 bound a common set of mRNAs in the brain. Reduced Mov10 in murine brain resulted in anxiety and increased activity in a novel environment, supporting its important role in the development of normal brain circuitry.

**Conclusions:**

Mov10 is essential for normal neuronal development and brain function. Mov10 preferentially binds RNAs involved in actin binding, neuronal projection, and cytoskeleton. This is a completely new and critically important function for Mov10 in neuronal development and establishes a precedent for Mov10 being an important candidate in neurological disorders that have underlying cytoarchitectural causes like autism and Alzheimer’s disease.

**Electronic supplementary material:**

The online version of this article (doi:10.1186/s12915-017-0387-1) contains supplementary material, which is available to authorized users.

## Background

Moloney leukemia virus 10 (Mov10) is a superfamily 1 (SF1) RNA helicase that binds to G-rich secondary structures and unwinds RNA in a 5′-to-3′ direction in an ATP-dependent manner [1, 2]. Mov10 was originally described as associating with RNA-induced silencing complex (RISC) factor Argonaute 2 (Ago2) and was required in microRNA (miRNA)-guided cleavage of a reporter [3]. Mov10 also has roles in nonsense-mediated decay, suppression of viral RNAs, and retrotransposition in cultured cells [1, 4, 5]. We found that Mov10 associates with the fragile X mental retardation protein (Fmrp) in the adult brain to regulate translation of a commonly bound set of RNAs by modulating their association with Ago2 [2]. Fmrp is required for normal cognition, and our findings suggested a possible role for Mov10 in brain function. Currently, there are no studies describing a role for Mov10 in the developing brain.

In the central nervous system (CNS), RNA helicases function by affecting neuronal differentiation, RNA localization, cell morphology, and apoptosis [6]. Examples of helicases that are miRNA-related include DHX36, which is required for dendritic localization of pre-miR134 [7], and DDX6, which binds TRIM32 to increase the activity of RISC [8]. Importantly, none of these helicases could functionally compensate for Mov10, since the Mov10 knockout is embryonic lethal in the mouse.

Mov10 is also significantly elevated in the brain shortly after birth through adolescence. Isolation of Mov10-associated RNAs from the P2 brain reveals two critical roles for Mov10 in early brain development: a suppressor of retrotransposition and a regulator of neuronal projections. Two-thirds of the Mov10-associated RNAs encode retroelements, including long interspersed nuclear elements (LINEs), while the rest of the messenger RNAs (mRNAs) encode proteins involved with neurite outgrowth and cytoskeleton.

Mov10 is a strong suppressor of endogenous transposition of L1, an active LINE element in cultured cells [5, 9]. During neuronal differentiation, there is increased L1 retrotransposition in the hippocampus and several regions of the adult brain. We hypothesize that Mov10 is elevated in the postnatal brain to suppress retrotransposition, which is highly active during this time in the brain [10]. As neurons mature and arborize, Mov10 regulates the translation of actin binding proteins and cytoskeleton, which is required for neuronal migration and function. This is the first study to show a role for Mov10 during embryogenesis and in postnatal brain development and function. We propose that Mov10 is vital for viability and for normal CNS development and function.

## Results

### Mov10 is elevated in the postnatal mouse brain

**Fig. 1:**
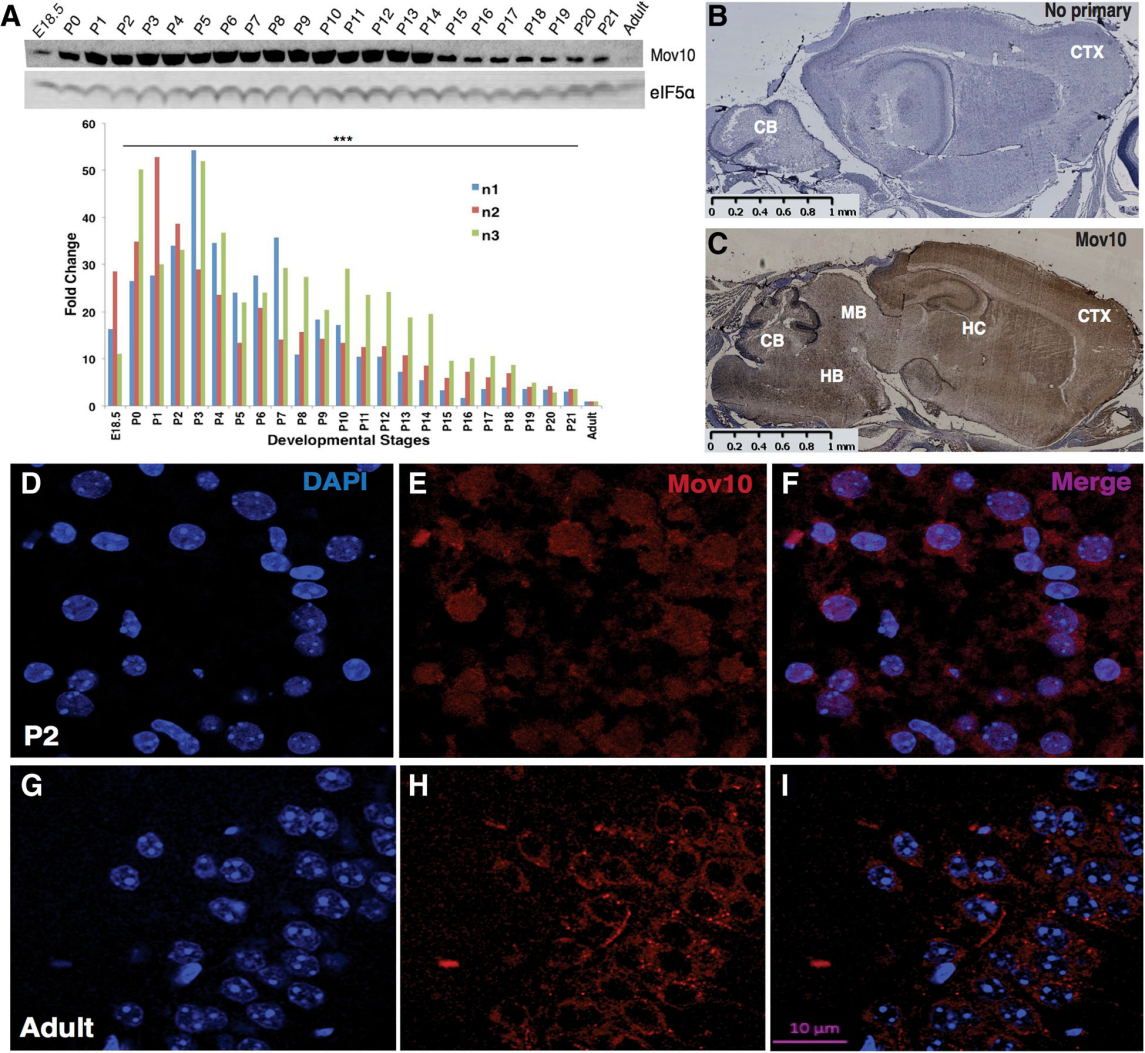
Mov10 is significantly elevated in young mouse brain and is both nuclear and cytoplasmic. **a** Brain extract (25 μg) from C57BL/6 at ages indicated was immunoblotted for Mov10 and eIF5α as a loading control (*top panel*). Bar graph of the three independent experiments is shown in the *bottom panel*. Spearman’s rank-order correlation (ρ (70) = –0.371, ***p = 0.001). **b** and **c** 3,3′-Diaminobenzidine (*DAB*) stain of P1 brain (sagittal section) counterstained with hematoxylin. *CTX* cortex, *HC* hippocampus, *CB* cerebellum, *HB* hindbrain, *MB* midbrain. **b** No primary antibody; **c** Mov10 antibody. Images obtained using the Hamamatsu NanoZoomer slide scanning system. Scale bar = 1 mm. **d**–**i** Mov10 immunohistochemistry of P2 brain (**d**–**f**) and adult hippocampus (**g**–**i**). Scale bar = 10 μm

Since Mov10 functionally associates with Fmrp [2], we examined Mov10 expression in the postnatal murine brain across development. As early as embryonic day 18, there was a higher level of Mov10 in the whole brain compared to adults (Fig. 1a, compare first and last lanes). Mov10 expression continued to rise at birth (P0) and remained elevated over adult levels until P10–P14, when it began to decline (Fig. 1a). We observed the same increase in postnatal Mov10 levels in a different mouse strain (Friend virus B-type (FVB), Additional file 1A), and it was independent of sex (Additional file 1B). We conclude that Mov10 is elevated in the postnatal and juvenile mouse brain, suggesting an important role for Mov10 in the developing brain.

To determine the pattern of Mov10 expression, we stained sagittal sections of postnatal and adult brain to examine if Mov10 was elevated in specific brain regions. Mov10 was highly expressed throughout the P1 brain, including the cortex, hippocampus, cerebellum, midbrain, and hindbrain (Fig. 1c). In contrast, there was very little Mov10 expression in the adult brain except in the hippocampus (Additional file 2A, right). However, the hippocampus and cortex of P0 mice expressed much more Mov10 than did the adult hippocampus and cortex (Additional file 2A, B). In addition, neurons appeared to have both nuclear and cytoplasmic staining in the P0 mice compared to the adult (Additional file 2A, see inset).

Since Mov10 has previously been described as cytoplasmic in both cultured cells [3, 5] and in cultured hippocampal neurons [11], we examined Mov10 localization in the P2 brain. We observed Mov10 in the nucleus as well as the cytoplasm (Fig. 1d–f, P2). In contrast, Mov10 was primarily cytoplasmic in the adult hippocampus (Fig. 1g–i, Adult). To verify these age-dependent differences in the intracellular localization of Mov10 and using a different Mov10 antibody, we examined hippocampal neurons cultured from P0 mice. We found that Mov10 was distinctly nuclear in day in vitro (DIV) 1 neurons (Additional file 2C, DIV1) compared to DIV14 neurons, where it was primarily cytoplasmic (Additional file 2C, DIV14), as previously reported [11]. We further confirmed the nuclear presence of Mov10 by biochemical fractionation of P2 brain (Additional file 2E). Mov10 expression was also examined in testes, where it is highly expressed, and found to be cytoplasmic (Additional file 2D). We conclude that Mov10 is in the nucleus and the cytoplasm in the postnatal brain.

### Mov10 knockout is embryonic lethal

Mov10, like Fmrp, is expressed throughout the brain. In order to study the function of Mov10 at postnatal stages in the brain, we attempted to generate a Mov10 knockout mouse using an embryonic stem (ES) cell with one copy of Mov10 targeted by a gene trap vector (Additional file 3). After screening 156 pups from heterozygote crosses, we found no viable Mov10 knockouts (Table 1) and concluded with >95% confidence that the Mov10 knockout has an embryonic lethal phenotype [12]. To determine when Mov10 exerts its crucial effect, we genotyped embryos from E9.5 and E12.5 and failed to detect any Mov10 knockout embryos at these early stages (Table 1). Based on these data, we conclude that Mov10 is essential for embryonic development in the mouse.

**Table 1:** Number of pups from heterozygote mating

Wild type	Heterozygous	Homozygous	Total
44 (28%)	112 (72%)	0 (0%)	156 (100%)
Screening of embryos from E9.5			
5 (36%)	9 (64%)	0 (0%)	14 (100%)
Screening of embryos from E12.5			
0 (0%)	21 (100%)	0 (0%)	21 (100%)

### Mov10 suppresses LINE retrotransposition in the nucleus

**Fig. 2:**
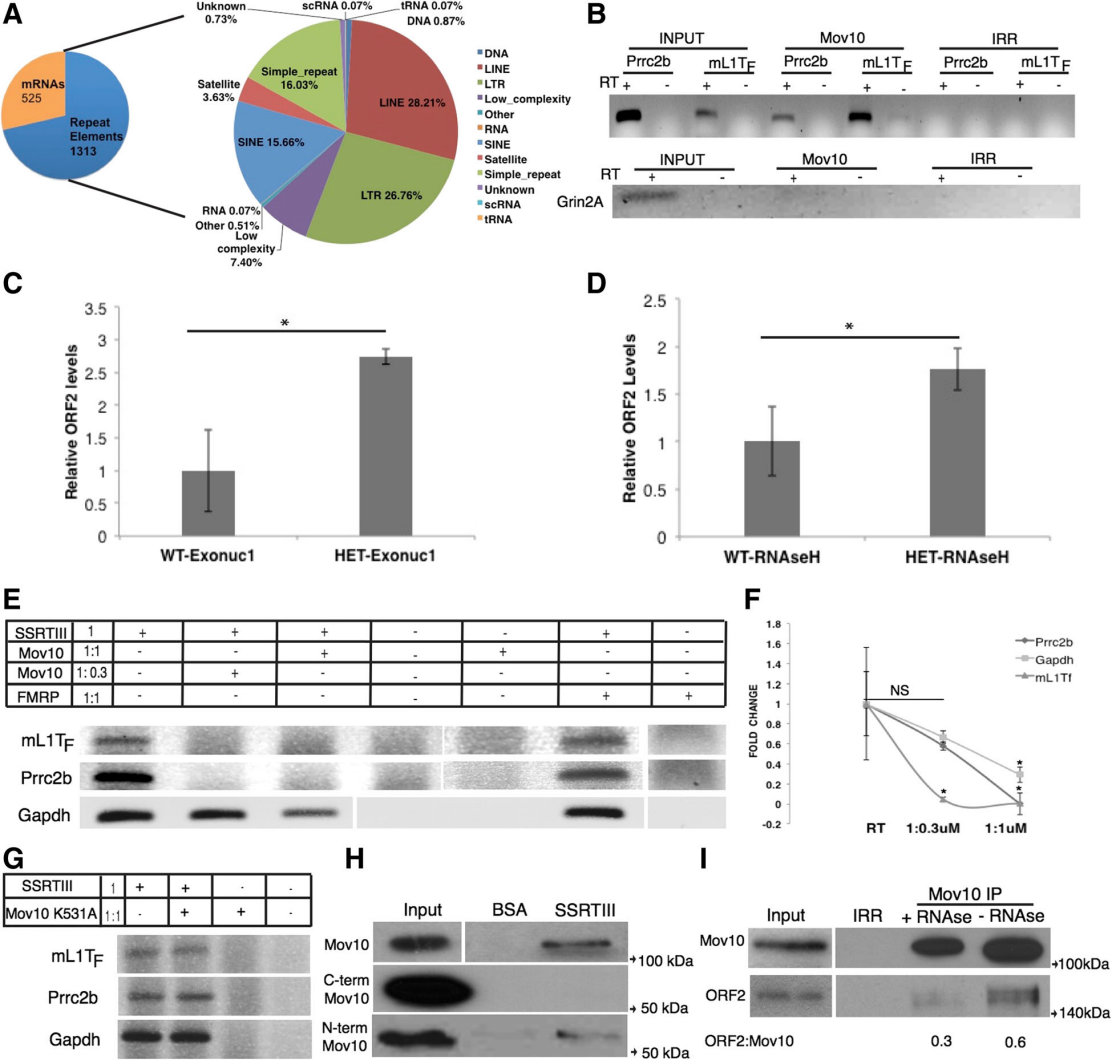
Mov10 binds repeat element RNA and mRNA in P2 brain and blocks retrotransposition. **a** Results of RIP followed by sequencing. *Left pie chart* shows the distribution of all immunoprecipitated RNAs. *Right pie chart* shows the classification of the repeat elements. **b** RT-PCR of Mov10 or irrelevant (*IRR*) IP from P2 brains for Mov10 individual nucleotide cross-linking immunoprecipitation (*iCLIP*) target mRNA (*Prrc2b*) and an active mouse L1 RNA (*mL1T*_*F*_) and for the mRNA *Grin2A*, which does not bind Mov10 [2]. **c**, **d** q-PCR of DNAse I- and RNAse H-treated genomic DNA isolated from P3 heterozygote (*HET*) or WT littermate brain (*n* = 3) amplified with ORF2 primers and 5S rDNA for normalization. Values plotted relative to adult genomic DNA content. Error bars represent standard error of the mean (*SEM*), *p<0.05 (Student’s *t* test, two-tailed). **e** Representative gel images of the reverse transcriptase assay set up as shown in the table; SuperScript III Reverse Transcriptase (*SSRTIII*) was preincubated with the indicated concentrations of purified Mov10 or human purified recombinant Fmrp as a control, followed by RT-PCR of RNAs bound by Mov10 (*Prrc2b* or *mL1T*_*F*_) or not (*Gapdh*). **f** RT-qPCR of Prrc2b, mL1T_F_, and Gapdh with indicated ratios of Mov10 and SSRTIII. Biological replicates are shown, and the fold change was compared to the RT-only reaction of each gene. Error bars represent standard deviation (*SD*), *p < 0.05 (Student’s *t* test, two-tailed). **g** Representative gel images for the RT assay using equimolar amounts of the Mov10 helicase-deficient mutant and SSRTIII. **h** Capture assay with WT, C-terminal and N-terminal of Mov10, and SSRTIII or bovine serum albumin (*BSA*) covalently coupled to beads. **i** Mov10 or IRR IP from P2 brains immunoblotted for L1-ORF2 (representative image of *n* = 3). The averages from biological replicates of the ratio between ORF2 and Mov10 for each lane are indicated below, the *p* values of which are not significant

To investigate the role of Mov10 in early brain development, we performed RNA immunoprecipitation (RIP) from P2 brains and sequenced the RNAs bound to Mov10. The total number of reads was 98,884,367; the number of aligned reads was 71,522,027 (74.59% aligned), and the number of uniquely aligned reads was 57,005,129 (59.45%). We used RIPSeeker to identify RNAs significantly enriched over input RNA [13] and found 2996 RIP peaks: 1313 overlapped with repeat elements from the long terminal repeat (LTR) family, the autonomous non-LTR family of LINEs, and the non-autonomous short interspersed nuclear elements (SINEs) in the RepeatMasker database (Fig. 2a, Additional file 4), and 525 peaks overlapped with RefSeq, indicating that they were mRNAs (Fig. 2a, Additional file 5). We validated the RIPSeeker result by immunoprecipitating Mov10 from the P2 mouse brain and performing reverse transcription-polymerase chain reaction (RT-PCR) on an endogenously active autonomous retrotransposon mL1T_F_ as well as Prrc2b, a brain mRNA target of Mov10 (Fig. 2b) [14]. Although Mov10 has previously been shown to bind the L1 transcript [5], we showed here that it binds L1 transcripts from the retrotransposition-competent T_F_ subfamily of mouse L1s (Fig. 2b). These primers have been used before by others [15]; however, it is possible that the RT-PCR to detect L1 expression is off-targeting to L1 sequence fragments that might be contained in mRNAs. Thus, we cannot rule out the possibility that some of the immunoprecipitated signal could be due to the presence of an mRNA that happens to contain the L1 primer target sequence.

Our hypothesis is that Mov10 binds the RNA of retroelements and inhibits their transposition in the developing brain. To test this hypothesis, we quantified the amount of genomic L1 in P2 brains from heterozygous Mov10 knockout mice compared to WT, hypothesizing that the reduction in Mov10 would lead to an increase in L1 retrotransposition events, as observed in MeCP2 knockout mice [16]. The qPCR was done using genomic DNA treated with exonuclease 1 to remove any unintegrated complementary DNA (cDNA) and RNAse H to remove DNA-RNA hybrids that might artificially contribute to the observed increase in LINE content. Similar to the MeCP2 study, we found a twofold increase in L1 genomic content in the Mov10 heterozygotes (Fig. 2c, d, Additional file 6), supporting a role for Mov10 in L1 suppression in the developing brain.

APOBEC3G is an RNA editing enzyme that plays a key role in regulating retrotransposition by directly binding reverse transcriptases [17, 18] and also by binding RNAs to sterically block reverse transcriptase (RT) activity [19, 20]. To determine whether Mov10 was able to block RT activity, we incubated equal molar amounts of Mov10 and SuperScript III Reverse Transcriptase (SSRTIII), an engineered version of M-MLV RT, and then performed a reverse transcription reaction in which total RNA from P2 brains was added. Reverse transcription of both L1 RNA and Prrc2b RNA was blocked by the addition of Mov10. In contrast, reverse transcription of the Gapdh transcript, which is not bound by Mov10 [2], was only partially inhibited (Fig. 2e, f, Additional file 6). We also tested purified recombinant human Fmrp, another RNA binding protein, in this assay and found that the addition of Fmrp did not have an effect on cDNA synthesis (Fig. 2e). Thus, Mov10 blocked reverse transcription of its bound targets more efficiently than that of non-target RNAs. Our data agree with the results of [5], where the researchers used the L1 element amplification protocol (LEAP) assay [21] to measure the ability of purified L1 RNP to reverse transcribe the bound L1 RNA. Over-expression of Mov10 in transfected cells inhibited reverse transcription of L1 in this assay [5]. However, another study used the LEAP assay with recombinant Mov10 (from OriGene) and found that reverse transcription was not suppressed [22]. These contradictory results could be due to the differing sources of Mov10 used.

Li and colleagues concluded that Mov10 blocks retrotransposition by facilitating L1 RNA degradation and its helicase activity is required for this function [9]. We tested the helicase-deficient mutant of Mov10, where a conserved lysine in motif I has been mutated to alanine [1], in our in vitro assay and found that it does not suppress the cDNA synthesis of either mL1T_F_ or Prrc2b (Fig. 2f, Additional file 6). Thus, the helicase function of Mov10 is required to block RT activity.

To determine if Mov10 directly binds RT, we coupled either SSRTIII or bovine serum albumin (BSA) to beads and found that only SSRTIII efficiently captured Mov10. Additionally, we performed the capture using either the C-terminal half or the N-terminal half of Mov10 and found that only the N-terminal half could bind SSRTIII (Fig. 2h). The unstructured N-terminus of Mov10 has been implicated in inhibiting HIV infectivity, though the exact mechanism is unclear [23]. Our data suggest that Mov10 binds reverse transcriptase through its N-terminal region and unwinds the L1 RNA using its C-terminal helicase domains. Importantly, Mov10 directly bound ORF2p, which is the RT/endonuclease encoded by L1 (Fig. 2i). RNAse treatment did not significantly change the amount of immunoprecipitated ORF2p (the difference between the indicated ratios is not significant), suggesting a protein-protein interaction. We conclude that Mov10 is elevated in the nucleus during postnatal brain development when retrotransposition is active to bind retroelement RNAs and block reverse transcription, which is a critical step for retrotransposon insertion.

### Mov10 associates with cytoskeletal mRNAs to regulate neurite outgrowth

**Fig. 3:**
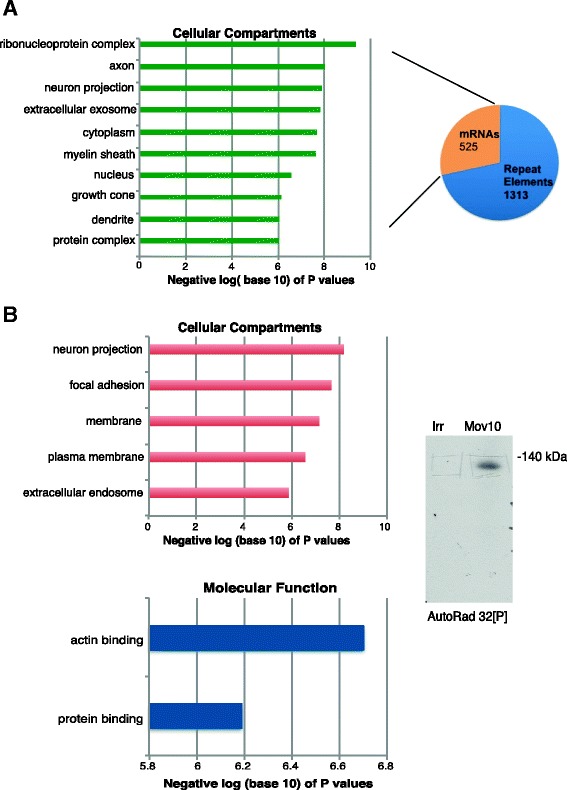
Mov10 binds mRNAs encoding proteins involved in neuron projection and cytoskeleton by RIP, iCLIP. **a** GO analysis of RIP mRNAs from postnatal brain. *Y axis*: GO terms for Cellular Compartment; *X axis*: negative log (base 10) of the ten lowest *p* values (see Additional file 5). **b** GO analysis of iCLIP mRNAs from postnatal brain. *Y axis*: GO terms for Cellular Compartment and Molecular Function; *X axis*: negative log (base 10) of the *p* values, showing only terms with *p* values of 10^–6^ or lower. *Right*: Autoradiograph of Irr and Mov10 IP from P0/P1 brains from iCLIP (see Additional file 7)

Approximately one-third of the Mov10-associated RNAs in the postnatal brain RIP-seq were mRNAs (Fig. 2a). We used the DAVID Bioinformatics database to analyze the Gene Ontology (GO) terms assigned to the 525 RNAs, which revealed axon, neuron projection, growth cone, and dendrite among the most significant categories (Fig. 3a, *p* values 9.1 × 10^–9^, 1.3 × 10^–8^, 7.8 × 10^–7^, 8.6 × 10^–7^ respectively). To independently verify this result, we performed individual nucleotide cross-linking immunoprecipitation (iCLIP) on brains isolated from P0–P1 mice (Fig. 3b, right panel) and obtained 92,798,446 reads; after quality trimming and deduplication, there were 5,269,506 reads. Further analysis revealed 61,471 unique tags present in the Mov10 IP compared to 3545 tags in the irrelevant IP. A total of 2988 of the tags aligned to the genome, 2333 were uniquely aligned, and 729 regions were identified. The gene identities are provided in Additional file 7. GO analysis using a P1 brain transcriptome as background revealed that RNAs encoding proteins involved in neuron projection had the lowest *p* value (Fig. 3b, Cellular Compartments category). Under GO category Molecular Function, actin binding and protein binding were the most enriched (Fig. 3b bottom). In addition, the GO term in the Biological Process category with the lowest *p* value (9.1 × 10^–5^) was actin cytoskeletal organization. These data suggest a cytoplasmic role for Mov10 in regulating actin and cytoskeletal mRNA expression in the postnatal brain.

**Fig. 4:**
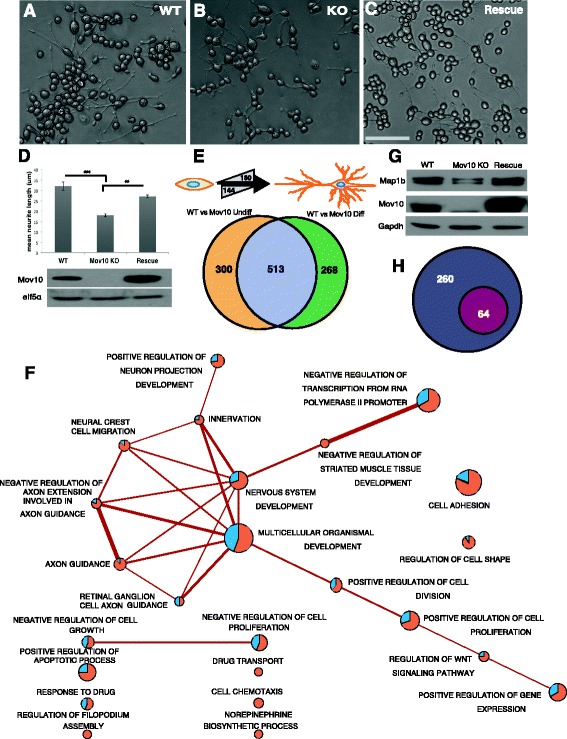
Mov10 is required for neurite outgrowth and neuronal morphology. **a**–**c** Brightfield images of N2a (*WT*), Mov10 knockout N2a (*KO*), and Mov10 transgene rescue of KO (*Rescue*). Scale bar represents 200 μm. **d** Quantification of neurite length of WT, KO, and Rescue, analyzed by one-way analysis of variance (*ANOVA*) (*F* (2,19) = 32, *p* = 0.000, *p* values, ** 0.01, *** 1.60484E-06). Error bars represent SEM. There were 500–800 differentiated neurons counted from triplicate experiments, and a total of 11 images were counted per condition. *Lower panel* is Mov10 immunoblot of WT, KO, and Rescue; eIF5α is loading control. **e** Schematic of significantly changed genes between WT undifferentiated and differentiated N2a. The number of differentially expressed genes as determined by Cuffdiff (*p* value <0.005 and fragments per kilobase million, *FPKM* >1) under both conditions is displayed (*top*). Venn diagram of genes differentially expressed in the KO versus WT. *Orange* (813): genes identified from comparison between undifferentiated WT and KO; *green* (781): genes identified from comparison between differentiated WT and KO; *purple* (513): Mov10-regulated genes (*bottom*). **f** Enrichment map of top Gene Ontology terms for the 513 Mov10-regulated genes (DAVID, *p* value <0.025) showing enrichment for genes related to nervous system development, axon guidance, and neuron projection. Fraction of genes up- (*blue*) and down- (*orange*) regulated in KO cells. **g** Microtubule-associated protein 1b (*Map1b*) immunoblot from WT, Mov10 KO, and rescue. *Gapdh* is the loading control. **h** Significantly changed genes between WT differentiated and undifferentiated (324). Of those genes, 64 significantly changed in the opposite direction of WT in the KO

To determine if Mov10 functions in neurite outgrowth, we used Clustered regularly interspaced short palindromic repeats (CRISPR)-Cas9 to knock out Mov10 (Additional file 8A) in cells of Neuro2a (N2a), a murine neuroblastoma that has long branching processes when grown on a substrate [24]. We induced differentiation of the WT and Mov10 knockout (KO) N2a cells and found significantly reduced neurite length in the Mov10 KO cell line compared to WT (Fig. 4a–d) that could be rescued upon re-introduction of the Mov10 transgene, suggesting that this phenotype was directly attributable to the loss of Mov10 and not an off-target effect.

To determine how Mov10 participates in differentiation, we isolated RNA from undifferentiated and differentiated WT and KO N2a cells and performed high-resolution RNA-seq analysis. Samples were sequenced extremely deeply to around 350,000,000 paired-end reads 100 base pairs (bp) long to capture lowly expressed genes, with at least 86% reads mapping to the mm10 mouse genome. We identified 16,551 genes and found that 324 genes changed significantly between the differentiated and undifferentiated states of WT N2a with 180 increasing and 144 decreasing (Fig. 4e top, Additional file 9). GO analysis revealed that RNAs implicated in cell cycle arrest were significantly changed (*p* value 4 × 10^–4^) under the GO category Biological Process, which is expected since undifferentiated cells proliferate in contrast to differentiated cells (Additional file 9). GO terms in the category Cellular Compartments revealed a significant enrichment for RNAs implicated in extracellular region/space and neuron projection (Additional file 8B).

To identify Mov10-dependent genes, we compared undifferentiated WT to KO and found 813 significantly changed RNAs (300 + 513), while a comparison of differentiated WT to KO showed 781 RNAs (513 + 268) that were significantly changed (Fig. 4e). We found that 513 genes were shared, suggesting that their expression was regulated by Mov10 and independent of differentiation (Additional file 10). GO analysis of these Mov10 target genes revealed strong clustering with terms relating to nervous system development, axon guidance, and neuron projection, with the majority of the genes in the groups being downregulated (Fig. 4f, orange indicates proportion significantly downregulated and blue indicates proportion significantly upregulated in the KO, see Additional file 10 for gene list). This result suggests a more general function for Mov10 as an RNA remodeler in addition to its role in revealing microRNA recognition elements (MREs). Similar to its function with Fmrp and Ago2, the fate of the mRNA depended on where Mov10 and Fmrp bound in the 3′untranslated region (3’UTR) [2, 25]. The data also show that Mov10 expression is critical for neurite outgrowth in N2a cells and is consistent with the data from mouse brain, where genes representing cytoskeletal components are the predominant functional categories. Additionally, we verified the Mov10 dependence of microtubule-associated protein 1b (Map1b), a cytoskeletal protein important in neurite outgrowth [26], and found that it was reproducibly reduced in the Mov10 KO and rescued on Mov10 re-expression (Fig. 4g). Map1b levels went down significantly in the KO cells under both differentiated and undifferentiated conditions, suggesting that it was a direct target of Mov10 irrespective of the differentiation program.

To identify the genes regulated by Mov10 that participate in differentiation, we compared the 324 (144 + 180) genes that significantly changed during WT differentiation (Fig. 4e) with Mov10 KO differentiation. There were 64 genes that significantly changed in the opposite direction in the differentiated Mov10 KO compared to WT (Fig. 4h). This group of 64 Mov10-dependent, differentiation-specific genes include key growth signals such as FGF1 and transcription factors like TEAD2 along with cytoskeletal genes such as actin isoforms and Tnnt1 (Additional file 9). We appreciate that the RNA-seq data include genes indirectly affected by Mov10 loss and are not necessarily directly bound by Mov10, although we do expect there to be some direct mRNA targets of Mov10. The direct binding of Mov10 to cytoskeletal mRNAs from the RIP and iCLIP data suggest that misregulation of those genes in the Mov10 KO leads to reduced neurite outgrowth. In fact some of the same cytoskeletal-related RNAs are found in both the Mov10-dependent genes in N2a (Additional file 10) and the mouse brain iCLIP lists (Additional Files 7 and 11﻿ as described in the Methods). We conclude that Mov10 plays a key role in neurite development and process formation through its regulation of cytoskeletal and neuroregulatory mRNAs.

**Fig. 5:**
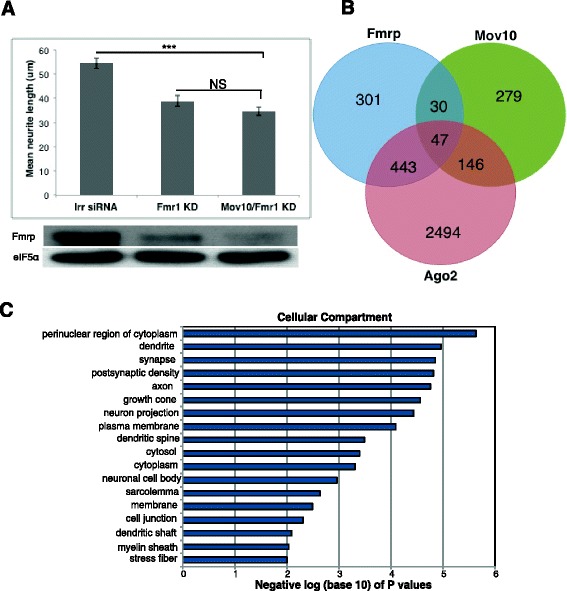
Mov10, Fmrp, and Ago2 bind mRNAs enriched for neuronal genes. **a** *Top panel* shows graph of mean neurite lengths between WT N2a cells treated with Irr small interfering RNA (*siRNA*), Fmr1 siRNA, and Mov10 KO N2a cells treated with Fmr1 siRNA (*n* = 3). Error bars represent SEM. Statistic: one-way ANOVA, (*F* (2,64) = 28, *p* < 0.001, *p* values = *** 6.01E-06, *NS* = 0.13). *Bottom panel* shows a representative Fmrp western blot of the three conditions. eIF5α is the loading control. **b** Venn diagram showing the overlap between brain-derived iCLIP targets of Fmrp, Mov10, and Ago2. All three proteins in the brain commonly bound 47 mRNAs, and the overlap was highly significant (see Methods). **c** GO analysis of the 47 overlapped genes from **b** *Y axis* is GO terms for Cellular Compartment; *X axis* is the negative log_10_ of the *p* values (see Additional file 12)

Fmrp predominantly binds brain mRNAs that function in neuron projection [27]. Because we have evidence that Fmrp and Mov10 functionally associate in HEK293 cells [2], we examined the effect of Fmrp knockdown on neurite outgrowth in N2a and found that it was significantly reduced (Fig. 5a). To ask whether Fmrp and Mov10 functioned in the same pathway, we compared neurite length in the Fmrp/Mov10 double knockdown to the Fmrp knockdown alone and found no difference (Fig. 5a). This result suggests that Fmrp and Mov10 function in the same pathway.

In earlier work, we showed that when Fmrp and Mov10 bound the same region in the 3′UTR of cobound mRNAs, binding by Ago2 was blocked [2]. In this subset of mRNAs, Fmrp-Mov10 interaction had a protective effect on the mRNA. To identify commonly bound brain mRNA targets of Fmrp, Mov10, and Ago2, we compared the iCLIP targets of whole brain-derived Mov10, analyzed as described (Methods and [2], Additional file 11), which came from postnatal mice (P0, P1). We compared these genes to previously published lists of iCLIP targets from brain-derived Fmrp [27], which came from mice aged P11–P25 and iCLIP targets from human brain-derived Ago2, which came from adult motor cortex and cingulate gyrus from males aged 44–68 [28] (Fig. 5b). Despite differences between species and age, we found significant overlaps between the Fmrp and Mov10 targets, between the Mov10 and Ago2 targets, between the Ago2 and Fmrp targets, and between all three proteins. All overlaps were highly significant (*p* = 2.15^–19^, *p* = 5.57^–26^, *p* = 4.85^–159^, and *p* = 0.00000 respectively). Using a permutation approach, we also determined that the amounts of overlap in the Venn diagram were significantly more than expected by chance (Fig. 5b) (see Methods). Thus, Fmrp, Mov10, and Ago2 bind a common set of brain mRNAs (Additional file 12). To understand what the functional consequences of such binding might be, we performed GO analysis of the 47 commonly bound Mov10-Fmrp-Ago mRNAs and found an enrichment of dendrite, synapse, and neuron projection terms under the GO category Cellular Compartments (Fig. 5c). These data suggest a miRNA-mediated function for cytosolic Mov10 in regulating cytoskeletal genes. *Map1b* was one of the genes present in the Fmrp-Mov10-Ago2 overlap (Fig. 5b) and is regulated by Fmrp through the miRNA pathway [29]. Similar to the fate described for the Fmrp/Mov10/Ago2-cobound mRNAs in HEK293, *Map1b* is reduced in the absence of Mov10 (Fig. 4g), suggesting a protective role for Mov10, likely in association with Fmrp.

### Role of Mov10 in neuronal maturation and behavior

**Fig. 6:**
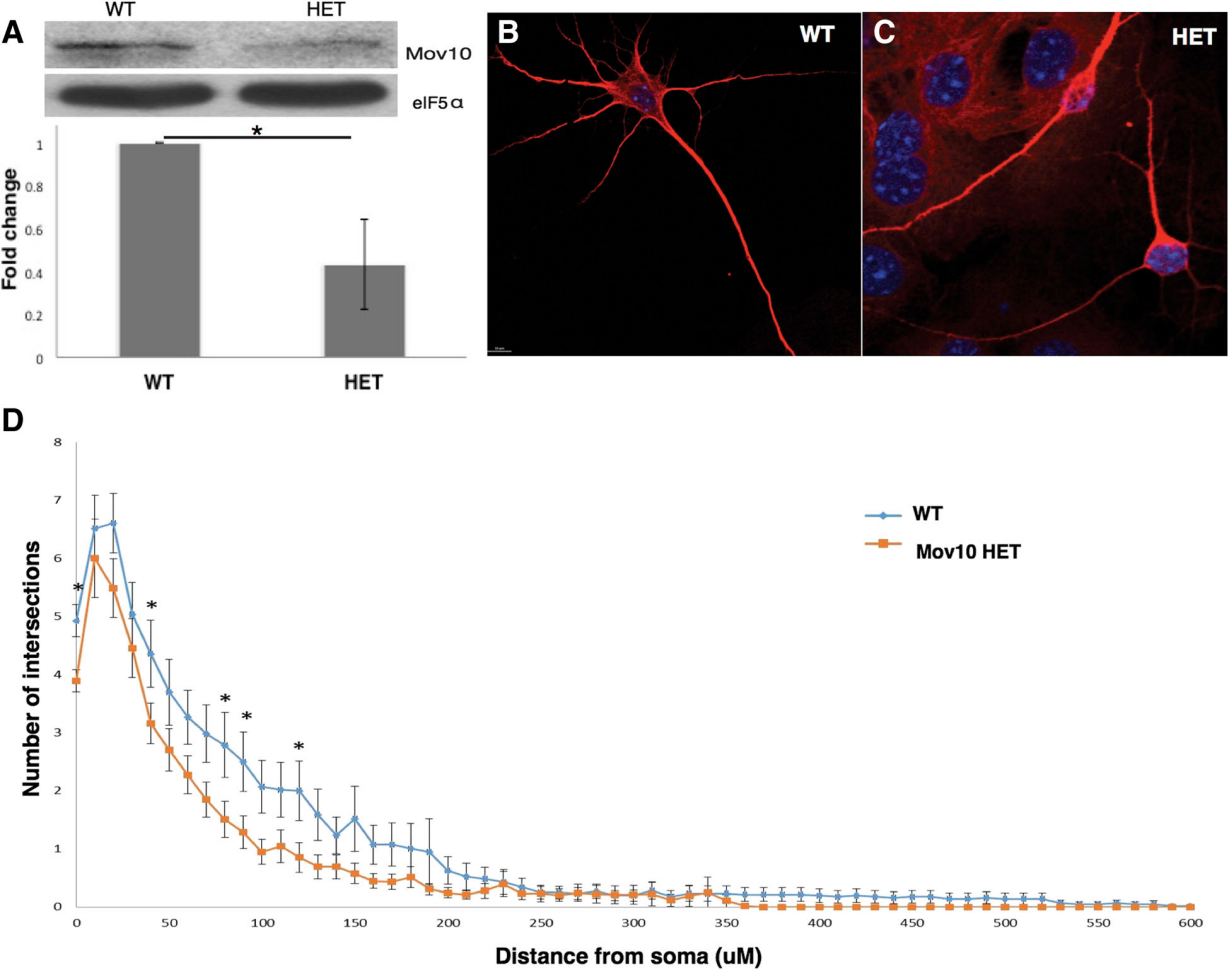
Normal levels of Mov10 are required for normal neuronal morphology. **a** 25 μg of total brain extract from P2 mice (genotypes shown above: HET is the Mov10 heterozygote, missing one copy of Mov10) immunoblotted for Mov10 and eIF5α. Immunoblot quantification (*n* = 3), error bars represent SD, * *p*< 0.05 (Student’s *t* test, two-tailed). **b** and **c** Map2 immunostaining of hippocampal neurons from DIV14 WT (**b**) and Mov10 heterozygous (**c**) neurons. **d** Dendritic morphology analysis. Confocal z-stacks of Map2-stained WT or Mov10 heterozygote DIV14 neurons were analyzed using Sholl. Statistics were calculated using two-way ANOVA followed by Bonferroni multiple comparisons test. Error bars indicate SEM and *p < 0.05. (*n* = 56 neurons for WT, and *n* = 94 neurons for Mov10 HET)

Because Mov10 is highly expressed in the developing brain and is required for normal neurite development, we hypothesized that the Mov10 heterozygote mouse would show a phenotype. This was the case for the microprocessor component DGCR8: loss of both alleles was embryonic lethal, but the heterozygotes had a neuronal and behavioral phenotype [30–32]. We verified that the Mov10 heterozygote mouse (HET) expressed half as much Mov10 in the brain (Fig. 6a) and then examined cultured hippocampal neurons from WT and Mov10 heterozygotes (HET). Mov10 heterozygotes had markedly less dendritic branching compared to the WT neurons (Fig. 6b, c). To quantify the difference between the Mov10 heterozygote and WT neurons, we performed Sholl analysis of all orders of branches (Total Sholl) [33, 34] and observed that a reduction in Mov10 levels significantly decreased dendritic branching at a maximum distance of 120 μM away from the cell body (*p < 0.05) (Fig. 6d). Thus, normal levels of Mov10 are required for normal dendritic arborization.

**Fig. 7:**
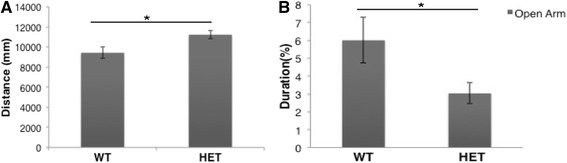
Normal levels of Mov10 are required for normal behavior. **a** Activity in an open field of WT and Mov10 heterozygous (*HET*) littermates (*n* = 17) plotted as distance traveled (millimeters). Error bars represent SEM, *p < 0.05 compared to WT (Student’s *t* test, two-tailed). **b** Percent time spent in open arms in the elevated plus maze by WT and Mov10 heterozygotes (*n* = 10). Both sexes were used because no significant difference was observed between sexes (WT, *p* = 0.71, Mov10 HET, *p* = 0.33; Student’s *t* test, two-tailed). Error bars represent SEM and *p < 0.05 compared to WT (Student’s *t* test, two-tailed)

To determine whether reduced Mov10 levels affected neuronal function, we tested the Mov10 heterozygotes in behavioral tests and found that the Mov10 heterozygote showed a significant increase in activity in an open field compared to WT littermates (Fig. 7a), suggesting anxiety and/or hyperactive behavior. The Mov10 heterozygotes also spent significantly less time in the open arms in an elevated plus maze test, suggesting an anxiety phenotype (Fig. 7b, Additional file 13B). In contrast, we did not see a difference in performance on the rotarod, trace fear conditioning, and novel object recognition (Additional file 13A, C, D, E). The increased activity in a novel environment and increased anxiety seen in the Mov10 heterozygotes suggests that an element of the neuronal circuitry is perturbed in these mice [35]. Thus, WT levels of Mov10 are required for normal neuronal development and function.

## Discussion

We show here two independent and previously undescribed roles for Mov10 in embryonic development and postnatal brain. Like the Ago2 knockout, the Mov10 knockout is also embryonic lethal [36–38], supporting their critical role in miRNA-mediated regulation during development. Since Mov10 is present in both the nucleus and cytoplasm in neurons, we believe that it is co-opted for critical but distinct functions in brain development. We propose that in addition to the cytoplasmic miRNA-mediated function of Mov10 in regulating neurite outgrowth in the brain, the developmentally timed increase of Mov10 acts as a defense against nuclear L1 retrotransposition.

### Nuclear Mov10 in LINE-1 suppression

There is extensive data in cell culture for Mov10′s role in suppressing LINE-1 retrotransposons [5, 9], although the mechanism is unknown. We present evidence that Mov10 directly binds retrotransposon mRNAs in the postnatal brain at stages when neuronal differentiation is high and acts to inhibit their reverse transcription (Fig. 2a, b) [39]. Significantly, the consequence of reducing Mov10 in the brain increases L1 content in the genome in P2 brains (Fig. 2c, d). The mechanism by which Mov10 inhibits reverse transcription could be by a steric block of ORF2p on L1 mRNA. The L1 endonuclease and reverse transcriptase ORF2p binds the poly(A) tract of L1 mRNA to mobilize it to the insertion site, where it nicks the DNA to prime reverse transcription in a 3′-to-5′ direction [40, 41]. We showed previously that Mov10 binds G-rich regions, including G-quadruplexes [2]. Thus, we suspect that Mov10 binds the G-rich polypyrimidine tracts [2] present in the 3′UTRs of L1 mRNAs [42] and also interacts with ORF2p through its N-terminal domain. Subsequently, Mov10 proceeds to unwind in the 5′-to-3′ direction, causing a steric hindrance to the progress of ORF2p. In support of this hypothesis, the helicase-deficient mutant of Mov10 is unable to block reverse transcription of L1 mRNAs (Fig. 2g). A recent study shows that the G-rich tracts in L1s stimulate retrotransposition [43]. We would hypothesize that Mov10 is elevated in the brain postnatally and localizes to the nucleus to suppress this event.

### Mov10 in neurite outgrowth, neuronal development, and brain function

The RIP from postnatal brain also shows a preponderance of actin and cytoskeletal mRNAs, suggesting an important role for Mov10 in regulating cytoskeletal dynamics in the brain (Fig. 3). The same observation was made in the RNA-seq analysis from Mov10 N2a KO cells, further confirming a critical role for Mov10 in neurite outgrowth (Fig. 4). We hypothesize that this reflects Mov10’s cytoplasmic role in miRNA-mediated regulation [2]. It likely plays a role with Fmrp in modulating Ago2 association with cobound RNAs (Fig. 5). Mov10 has low expression in the adult brain (Fig. 1), similar to what is reported in the Allen Brain Atlas [44, 45]. However, there is no report of Mov10 expression in the developing brain. We observe an approximately 40-fold increase in Mov10 levels in P0–P3 mouse brain (Fig. 1) when events like synaptogenesis, synaptic pruning, and neuronal differentiation are occurring to shape normal brain circuitry [39]. Mov10 is important for these events since a 50% reduction in Mov10 levels leads to less dendritic complexity in hippocampal neurons (Fig. 6b–d). These data suggest a role for Mov10 in the normal development of brain circuitry. Based on the evidence that Mov10 preferentially binds cytoskeletal mRNAs, we hypothesize that the reduction in Mov10 affects dendritic morphology and synaptic remodeling in the brain. Accordingly, the Mov10 heterozygote mice show increased activity in a novel environment and higher anxiety, suggesting that Mov10 is required for normal brain function (Fig. 7a, b). It is also possible that the increased retrotransposition activity in the Mov10 heterozygote could be contributing to the neuronal phenotype and behavior. In fact, increased L1 insertions have been implicated in the development or predisposition to psychiatric disorders [46, 47].

The cytoarchitecture of neurons has implications in the neuropathology of autism and neurodegenerative disorders [48, 49]. In fact, CNVs containing Mov10 have been found in individuals with developmental delay [50–53]. Our study demonstrates that Mov10 is essential in embryonic development, in normal neuronal development, and in brain function. It remains to be determined how Mov10 levels are regulated in the brain.

## Conclusions

Mov10 is significantly elevated in the postnatal murine brain, where it binds retroelement RNAs and mRNAs. Mov10 suppresses retroelements in the nucleus by directly inhibiting cDNA synthesis, while cytosolic Mov10 regulates cytoskeletal mRNAs to influence neurite outgrowth. Finally, reduced Mov10 in the murine brain results in anxiety and increased activity in a novel environment. In summary, Mov10 is essential for embryonic viability and for normal CNS development and function.

## Methods

### Western blot

Samples from at least three biological replicates were prepared for immunoblotting after quantification by Bradford assay and suspension in 1× sample buffer, resolved by SDS-PAGE and analyzed by western/immunoblotting. Briefly, membranes were blocked with 5% non-fat dry milk in phosphate-buffered saline (PBS) containing 1% TWEEN-20 for 1 h at room temperature. Primary antibody was applied for 1 h at room temperature or overnight at 4 °C followed by a brief wash in 1% non-fat milk PBS containing 1% TWEEN-20 wash buffer. Horseradish peroxidase (HRP)-conjugated secondary antibody was applied at 1:5000 dilution for 1 h at room temperature and washed 4 × 15 min using wash buffer. The HRP signal was detected using an enhanced chemiluminescent (ECL) substrate and exposed to film. The antibodies used were anti-Mov10 (A301-571A, RRID: AB-1040002; Bethyl Laboratories, Montgomery, TX, USA) at 1:1000, anti-Cbx7 (sc-70232, RRID:AB_2071502; Santa Cruz Biotechnology, Santa Cruz, CA, USA) at 1:2000, anti-eIF5 (RRID:AB_631427) (Santa Cruz) at 1:10,000, anti-Gapdh (ab9484, RRID:AB_307274; Abcam, Cambridge, UK), anti-LINE-1 (sc-67198, RRID:AB_1249550; Santa Cruz), and HRP-conjugated anti-rabbit and anti-mouse antibodies from GE Healthcare (RRID:AB_772191) and Jackson Immunoresearch, West Grove, PA, USA (RRID:AB_2338512) respectively. The level of significance and tests performed are described in the figure legends for each experiment.

### Whole mouse brain fixation, sectioning, and staining

Three adult C57BL6 (Envigo, USA [formerly known as “Harlan”]) males were euthanized and whole body fixation was done using 4% paraformaldehyde in PBS. The brain was dissected out and fixed with a series of ethanol washes for 30 min (25%, 50%, 70%, 83%, 95%, and 100%) and left in methyl salicylate for 3 h to overnight before embedding in paraffin. For P0 and P1 pups, whole, skinned heads were fixed in 4% paraformaldehyde overnight and dehydrated similar to adult brain. Sections were prepared using a Spencer 820 rotary microtome and dried overnight at room temperature. The sections were de-paraffinized using xylene and rehydrated through a series of ethanol washes (100% and 95% followed by 1× PBS) before boiling in 1× citrate (pH 6.0) for epitope retrieval. The sections were stained using a primary antibody to Mov10 (Abcam ab60132, RRID: AB_944250) at 1:100 and Alexa fluor 596 at 1:800 (RRID: AB_2340621, Jackson Immunoresearch) before imaging using a NanoZoomer Slide Scanner (Hamamatsu, Shizuoka, Japan) and Zeiss LSM700 confocal microscope. 3,3′-Diaminobenzidine (DAB) staining was done using the same antibodies and following the instructions in the DAB staining Kit (Vector Labs, Burlingame, CA, USA) and counterstained with hematoxylin before imaging on the NanoZoomer Slide Scanner. Testes sections were stained using anti-Mov10 (A500-009A, RRID: AB_10950563, Bethyl) and anti-mouse-Cy3 (RRID: AB_2340813, Jackson Immunoresearch).

### Brain IP, RT-PCR, and RNA sequencing

For the brain-RIP-seq, brains were harvested from 28 WT P2 pups. For confirming specific transcripts by brain IP, three WT P2 brains were used. All were triturated in Hank’s balanced salt solution (HBSS) and then UV cross-linked thrice for the confirmatory IP. Triturated cells were lysed in lysis buffer (50 mM Tris-Cl 7.5, 300 mM NaCl, 30 mM ethylenediaminetetraacetic acid (EDTA), 0.5% Triton), cleared by ultracentrifugation (35,000 rpm at 35 min at 4 °C), and sequentially immunoprecipitated with an irrelevant rabbit polyclonal antibody (anti-EGFP, Clontech, Mountain View, CA, USA; RRID: AB_10013427) followed by IP with Mov10 antibody (A301-571A, RRID: AB_1040002, Bethyl). Both IPs were washed sequentially for 10 min with lysis buffer and twice with wash buffer (1× PBS, 0.1% sodium dodecyl sulfate (SDS), 0.5% sodium deoxycholate, 1% NP40). The IPs were treated with 500 units of RNAse-free DNAse I, washed once for 10 min with high salt buffer (50 mM Tris, 1 M NaCl, 1 mM EDTA, 1% NP40, 0.1% SDS, 0.5% sodium deoxycholate). To isolate associated RNA, the IPs were treated with proteinase K followed by TRIzol (Ambion) extraction for RNA isolation. The ethanol-precipitated RNA was quantified, and equal amounts were used for cDNA synthesis followed by RNAse H treatment. The RNA was extracted with phenol-chloroform and precipitated in ethanol and converted into cDNA using Oligo dT primer and SuperScript III Reverse Transcriptase. qRT-PCR was performed with iQ SYBR Green Supermix (Bio-Rad Laboratories, Hercules, CA, USA) using a StepOnePlus RT PCR machine (Applied Biosystems) with gene-specific primers. For the brain IP-RNA sequencing, WT brains were homogenized in the same manner as described above but were not UV cross-linked. Additionally, the TRIzol-extracted RNA was cleaned using an RNA Clean & Concentrator Kit (Zymo Research, Irvine, CA, USA) before sequencing.

### RIP-seq analysis

Total input RNA and RNA extracted from the irrelevant IP and the Mov10 IP were used for making libraries and produced over 230 million reads with perfect quality scores. The contribution from the irrelevant IP was negligible and removed from further analysis. Each fastq file was broken into 100 smaller fastq files using a Perl script downloaded from [54]. TopHat2 (version 2.1.1, RRID: SCR_013035) was run on each individual smaller fastq file using –g 2000 and all default parameters. Setting –g 2000 instructs TopHat2 to allow up to 2000 alignments to the reference (version mm10 that is not masked for repetitive regions, obtained from Illumina iGenomes) for a given read.

The resulting alignment files in Binary Alignment/Map (BAM) format were merged into a single BAM file for each sample using samtools (version 1.3). BAM files were then sorted based on chromosome coordinates of alignments using novosort (novocraft version 3.02; RRID: SCR_014818). The total number of reads from the Mov10 IP was 98,884,367, the number of aligned reads was 71,522,027 (74.59% aligned), and the number of uniquely aligned reads was 57,005,129 (59.45%). From the input sample, the total number of reads was 67,566,885, and the number of aligned reads was 59,620,379 (88.24% aligned). The number of uniquely aligned reads was 29,124,706 (43.11%). To identify protein-associated transcripts, the Bioconductor-based statistical package RIPSeeker was used [13]. RIPSeeker’s (version 3.3; RRID: SCR_006810) function ripSeek was run using the alignments generated by TopHat2 with parameters:uniqueHit = TRUE, assignMultihits = TRUE, rerunWithDisambiguatedMultihits = TRUE, and binSize = NULL. Setting uniqueHit = TRUE requires training the hidden Markov model (HMM) with only the unique hits; assignMultihits = TRUE enables function disambiguateMultihits to assign each multihit to a unique locus based on the posterior probabilities derived from HMM; rerunWithDisambiguatedMultihits = TRUE tells RIPSeeker to retrain the HMM using the dataset with disambiguated multihits; binSize = NULL enables automatic bin size selection. ripSeek function was run separately for plus strand and minus strand, and the output files in GFF3 format were combined into a single GFF3 file that contains genomic coordinates for all the regions identified to be significantly enriched in Mov10 IP compared to Input. To identify repeat elements and transcripts that overlap with RIP regions identified by RIPSeeker, two tab-delimited text files were downloaded from the University of California, Santa Cruz (UCSC) Genome Browser’s table browser interface. One text file contains genomic coordinates of all repeat elements on mouse reference genome mm10 extracted from the RepeatMasker database, and another text file contains genomic coordinates of all mouse transcripts on mouse reference genome mm10 extracted from the National Center for Biotechnology Information (NCBI) RefSeq database (RRID: SCR_003496). Bedtools intersect (version 2.25.0) was run to identify repeat elements and mouse transcripts that overlap with RIP regions. A total of 2996 RIP peaks were identified: 1313 overlapped with repeat elements from the RepeatMasker database, and 1683 peaks had no overlaps with RepeatMasker with 755 peaks overlapping with RefSeq.

### Mouse brain iCLIP analyses

Sixteen brains from P0 and P1 mice were triturated in HBSS and UV cross-linked three times (using Stratalinker) with mixing between treatments. A published iCLIP protocol was followed [55, 56]. The irrelevant IP was performed with a rabbit affinity purified antibody EGFP (RRID: AB_10013427, Clontech) The Mov10 IP was performed with antibody (A301-571A, RRID: AB_1040002, Bethyl). Mov10-CLIP libraries were sequenced by the University of Illinois, Urbana-Champaign (UIUC) Core Sequencing Facility using the Illumina HiSeq2000 platform. The fastq data were trimmed using Trimmomatic (version 0.30, RRID: SCR_011848) to (1^st^) trim off (crop) the last 14 nucleotides from all the reads, (2^nd^) trim nucleotides with a quality value lower than 20, from the far (3′ end) of the read, (3^rd^) trim nucleotides with a quality value lower than 25 from the 5′ end of the read, and (4^th^) remove the adaptor/known contaminant. Reads with 30 or more nucleotides remaining after the trimming were kept. These data, processed as described below, are presented in Additional file 7. Reads with 18 or more nucleotides remaining after the additional trimming were kept and processed as described below. The genes identified are in Additional file 11 and were used in the comparisons in Fig. 5.

The fastq files were converted to fasta files, which were compressed to eliminate duplications, based on the tags. The compressed fasta file of tags was then separated into two files—representing the irrelevant and Mov10 immunoprecipitations—each file containing the tags with a specific barcode. This step utilized scripts that did the separation and also removed the barcode from the read, in preparation for the alignment step. The separated samples were aligned to the mouse genome (mm10) using Novoalign (RRID: SCR_014818). The only parameter specified was “-t 60”, this allows for two mismatches between the genome and the read. Uniquely mapping reads were extracted from the resulting sam files, and the information was converted to BAM format using samtools (samtools view; RRID: SCR_002105). The BAM format was converted to bed (genome interval) format using bedtools (version 2.17.0; RRID: SCR_006646) (bamToBed). The genome intervals of the reads (bed file) for each sample were merged into larger intervals using bedtools (mergeBed). The new interval/region is a location with a set of overlapping reads. Any regions that had any presence in the control Irrelevant samples (intersectBed) were removed to give the final set of experimental genome intervals that have reads mapping to them in the Mov10 immunoprecipitations, which will be referred to as “regions” from here on. IntersectBed (bedtools) was used to determine which regions overlap with genes, exons, UTRs, and lncRNAs (Ensembl) regions using bed files specific for these features respectively. All regions that overlap with genes, but not with exons, are considered intronic, and all other regions are considered intergenic. DAVID 6.8 analysis [57, 58] (RRID:SCR_001881) was performed on the Mov10 CLIP targets using a P1 C57Bl/6 brain background (Gene Expression Omnibus (GEO) numbers GSM417923, GSM417922, and GSM417921).

### In vitro reverse transcriptase assays

Total RNA from three P2 mice brains was used in the reverse transcription assay with either SuperScript III Reverse Transcriptase (Invitrogen ThermoScientific, Carlsbad, CA, USA) alone or with equimolar amounts of recombinant Mov10 or Fmrp [2] after pre-incubating the proteins on ice for 5 min. cDNA synthesis was carried out at 50 °C for 45 min, then 70 °C for 15 min. RT-PCR reactions was carried out on the cDNA samples using primers to Prrc2b, mL1T_F_, or Gapdh (Additional file 14). For qRT-PCR, iQ SYBR Green Supermix (Bio-Rad) was used, and the reactions were set up in a StepOnePlus RT PCR machine (Applied Biosystems) with gene-specific primers.

### Purification of the C-terminal, N-terminal, and Mov10 helicase mutant

The myc-tagged Mov10 helicase mutant was generated using site-directed mutagenesis to mutate the conserved lysine in motif I to alanine (K531A). The HA-tagged N-terminal half and C-terminal half plasmids were obtained from the source in Ref. [23]. Constructs were transfected using polyethylenimine (PEI, # 408727, Sigma) in FreeStyle HEK 293 F cells (Invitrogen) and cultured according to the manufacturer’s protocol and as described [2]. The cells were harvested after 48 h and lysed in lysis buffer (50 mM Tris-Cl 7.5, 150 mM NaCl, 30 mM EDTA, 0.5% Triton) containing protease inhibitors (Roche, Indianapolis, IN, USA) and spun at 14,000 rpm for 5 min at 4 °C. The supernatant was immunoprecipitated with anti-HA magnetic beads (Thermo Fisher Scientific, Carlsbad, CA, USA) for the HA-tagged C-terminal and N-terminal half plasmids of Mov10, and the peptide was eluted with HA peptide (2 mg/mL, Protein Sciences, Roy J Carver Biotech Center, UIUC) for 2 h at 4 °C. The Mov10 helicase mutant was immunoprecipitated using agarose beads coupled to myc antibody (RRID: AB_10109522, Sigma-Aldrich, St. Louis, MO, USA) and the peptide eluted using the myc peptide (2 mg/mL Protein Sciences). Protein concentrations were calculated using a Bradford assay (Bio-Rad Laboratories) and visualized on silver stain.

### Protein binding assay

For testing direct binding of recombinant WT and helicase-deficient Mov10 and SSRTIII, 5 μg of BSA or SSRTIII was coupled to M270-epoxy Dynabeads (Life Technologies, Carlsbad, CA, USA) overnight at 4 °C. The protein-coated beads were washed according to the manufacturer’s protocol, and 10 μL of beads (2.5 μg protein) was used in a reaction with equimolar amounts of recombinant Mov10 in three independent trials. The reactions were incubated on ice for 30 min and washed in 1× PBS. The samples were subsequently processed for western blotting with the Mov10-specific antibody.

### Genomic DNA isolation and qRT-PCR

Brains were dissected from three separate WT and Mov10 heterozygote mice, and the total genomic DNA was isolated using the DNAzol reagent (Invitrogen). The DNA was ethanol precipitated and treated with Exonuclease I (New England Biolabs (NEB), Ipswich, MA, USA) or RNAse H (NEB) as per the manufacturer’s protocol. Equal amounts were used in quantitative RT-PCR using primers for ORF2 and 5S rDNA to estimate total LINE-1 content (see Additional file 14).

### N2a transfection, neurite analysis, and preparation for RNA sequencing

N2a WT (RRID: CVCL_0470) and Mov10 KO clones were plated in triplicate at a density of 1.5 × 10^4^ cells per well and incubated for 24 h at 37^o^ C in Dulbecco’s modified Eagle’s medium (DMEM, with 10% fetal calf serum, FCS). One set of the Mov10 KO wells were transfected using Lipofectamine 2000 (Thermo Fisher Scientific) with a plasmid bearing full-length mouse Mov10 before differentiating with DMEM (2% FCS) and 20 μM retinoic acid (Sigma-Aldrich) a day later. Cells were allowed to differentiate for 48 h and imaged under transmitted light using an EVOS cell-imaging microscope. The images were anonymized and analyzed by an experimenter blinded to the conditions using the AxioVision image analysis software. About 500–800 differentiated neurons were counted from triplicate experiments, and a total of 11 images were counted per condition.

For total RNA sequencing, 2 × 10^5^ cells were plated in a 6-well plate and differentiated using 2% FCS and retinoic acid after 24 h. The cells were allowed to differentiate for 48 h with a media change every 24 h. Total RNA was isolated using TRIzol reagent (Ambion), and the RNA quality was checked on a 1% MOPS-agarose gel. The samples were DNAse treated and cleaned and concentrated using the RNA Clean & Concentrator Kit (Zymo Research) before sequencing.

### RNA-seq analysis of N2a

The Illumina HiSeq4000 sequencer was used in paired-end mode. Library adapters were trimmed and reads were mapped to the Encyclopedia of DNA Elements (ENCODE) mm10 genome using Spliced Transcripts Alignment to a Reference (STAR) in paired-end mode [59]. Each sample produced ~350,000,000 million paired reads 100 bp in length. These reads mapped to the genome with >85% coverage. Cuffdiff was used to identify differential expression between experimental groups [60]. Cuffdiff results were further filtered by *p* value (<0.005), expression levels (one or both conditions contains the gene at >1 fragment per kilobase million, FPKM), and fold change (log_2_ (fold change) >1). RRID numbers for the software are as follows: Cuffdiff: SCR_001647; CummeRbund: SCR_014568; DAVID: SCR_001881; Cytoscape: SCR_003032.

### GO analysis

Gene lists generated from RNA-seq analysis were analyzed for patterns in Gene Ontology using DAVID 6.8 [57, 58]. An enrichment map of significant ontology terms was generated using the Cytoscape plugin EnrichmentMaps [61, 62].

### CRISPR-Cas9 knockdown in N2a cells

Guide RNAs (Additional file 14) were designed to the mouse Mov10 locus as described in [63] and cloned into pX459 plasmid (Addgene, Cambridge, MA, USA). Constructs were transfected into WT N2a, serially diluted into 96-well plates, and grown under puromycin (2 μg/mL) selection. Puromycin-resistant colonies were selected and screened for Mov10 expression using western blot analysis and confirmed by sequencing.

### Nuclei purification and fractionation

WT brain tissue was extracted from three P2 mice, and the nuclei were prepared as described in [64]. We minced 100 mg of the tissue and extracted the nuclei using a nuclei isolation kit (Sigma NUC201), separated by ultracentrifugation at 40,000 rpm for 30 min at 4 °C (Beckman TL-100). Nuclei were suspended in cytoskeletal (CSK) buffer containing 10 mM piperazine-*N*,*N*′-bis(2-ethanesulfonic acid) (PIPES), 300 mM sucrose, 1 mM ethylene glycol tetraacetic acid (EGTA), 200 mM NaCl, 1 mM dithiothreitol (DTT), and Roche protease inhibitor cocktail. The separated nuclei containing CSK buffers were supplemented with 1 mM phenylmethylsulfonyl fluoride (PMSF), left at 4 °C for 5 min, and centrifuged at 2000 × g for 5 min at 4 °C to separate the nucleoplasmic and chromatin fractions.

### Hippocampal neuron culture

Mov10 heterozygotes were genotyped at P0 using tail samples, and DNA was extracted with the KAPA Fast Extract Kit (# KK7103, KAPA Biosystems, Wilmington, MA, USA). After genotyping, mouse hippocampi were dissected and cultured on embryonic day 20 (E20), or postnatal day 0 (P0), as described [65]. Coverslips were coated overnight with poly-l-lysine (Sigma, P4707, 10 μg/mL) and 10^5^ cells/well were plated for immunofluorescence (IF) in minimum essential medium (MEM) supplemented with 10% fetal bovine serum (FBS). After 24 h, the medium was switched to Neurobasal (NB) medium (Gibco, 21103049) supplemented with B-27 (Gibco, 17504-044). Half of the media was removed and replaced with fresh NB medium every 3 days.

### Immunofluorescence and microscopy of cultured neurons

Neurons grown on coverslips were fixed in 4% paraformaldehyde for 10 min at room temperature, either 24 h after initial culture (DIV0) or 14 days later (DIV14). Samples were blocked in 10% normal donkey serum (Jackson ImmunoResearch, 017-000-121) for 30 min at room temperature. Mov10 primary rabbit polyclonal antibody (1:1000, Bethyl, A301-571A RRID: AB_1040002) or MAP2 antibody (1:1000 dilution, Millipore # AB5622, RRID: AB_91939) was incubated overnight at 4 °C. Secondary antibody (Alexa 594 goat anti-rabbit [1:4000, Jackson ImmunoResearch, 111-585-144, RRID:AB_2307325]) was added for 2 h at room temperature. Coverslips were inverted unto glass slides containing mounting media with 1 μg/mL 4′,6-diamidino-2-phenylindole (DAPI). Fluorescence images of DIV0 and DIV14 neurons were obtained with a Zeiss LSM 700 inverted confocal microscope using a 40× and 63× EC Plan-Neufluar 1.30 oil objective respectively. Images were captured with a cooled charge-coupled device (CCD) camera running Zen 2012 software. A total of 10–15 0.2-μM-thick sections were acquired as z-stacks for each time point.

### Sholl analysis

Sholl analysis of all orders of branches (Total Sholl) was performed. Confocal z-stacks of either WT or Mov10 heterozygous DIV14 neurons immunostained for MAP2 were imported into ImageJ. A dendritic complexity analysis, including Sholl analysis, was performed according to the protocol described [33].

### Behavior tests

The sample size for behavioral testing was estimated using G^*^ power [66] from a pilot study using 5 animals. The analysis recommended a sample size of 4 animals per group and showed an effect size *d* = 2.522797 and a power of 0.842302. We decided to use at least 10–15 animals per group to account for attrition and outliers. We excluded outliers based on a z-test cutoff of +/– 2 standard deviations from the mean. Mice aged 60–65 days old were tested at the same hour of the day in the following sequence: open field, novel object recognition, rotarod, elevated plus maze, and trace fear conditioning. The experimenter was blinded to the genotypes. Both sexes were tested on the rotarod, elevated plus maze, and trace fear conditioning.

#### Open field test

The test was performed on the first day of the novel object recognition test. Mice were exposed for 5 min to a rectangular arena (46 × 25 × 20 cm), and the distance covered was tracked using a Logitech HD Pro webcam. The videos were then analyzed using the TopScan LITE software (Cleversys Inc., Reston, VA, USA).

#### Novel object recognition

The test was performed as described [67]. Briefly, mice were habituated to the empty arena on the first day for 5 min. After 24 h, two similar objects were presented, and the interaction with each object was tracked using a webcam. The pair of objects used in the test was randomized between animals. On day 3, a novel object replaced one of the objects, and the mice were video-tracked. The placement of the novel object was randomized between animals. The videos were analyzed using the ObjectScan software (Cleversys Inc.) to estimate interaction times.

#### Rotarod

Mice were placed on a stationary rotarod (AccuRotor Rota Rod Tall Unit, 63-cm fall height, 30-mm diameter rotating dowel; Accuscan, Columbus, OH, USA). The dowel was then accelerated at 60 rpm/min, and the latency to fall (in seconds) was recorded. The procedure was repeated for four consecutive trials, which were averaged to give the daily latency to fall for each mouse. The trials were repeated for 2 more days for a total of 3 days.

#### Elevated plus maze

The apparatus consists of four arms (66 × 6.4 cm), an open area in the center (6.4 cm), two opposing open arms, and two opposing closed arms (20-cm-high wall) with sliding doors at the end. The maze is elevated at a height of 60 cm from the floor. Mice were placed in the center of the maze, and the time spent in each zone was recorded for 10 min using the webcam. The TopScan LITE software analyzed the videos.

#### Trace fear conditioning

A modified procedure of the test was performed as described [68]. Mice were trained by exposing them for 3 min to a chamber (34 × 28 × 30 cm) where they received three consecutive pairs of tone (10 s) and shock (0.5 mA, 1 s) with an empty trace interval of 1 s and a 3-min break between each tone-shock pairing. Behavior was recorded using a webcam in a new context with the same tone but without shock 48 h after training (trace fear conditioning). Five days after training, the mice were placed back in the original training chamber without tone or shock and recorded for 6 min (context conditioning). The videos were analyzed using TopScan LITE to measure the level of freezing in both cases.

### Venn diagram for Mov10/Fmrp/Ago2 overlaps

A total of 842 genes with Fmrp-CLIP sites from [27] contained NCBI’s Entrez Gene IDs in the https://www.ncbi.nlm.nih.gov/pmc/articles/PMC3232425/bin/NIHMS314927-supplement-Suppl_Table_S2A-C.xls. All but two of the IDs were in the current Bioconductor org.Mm.eg.db database (version 3.4.0) (RRID: SCR_006442). According to the NCBI, these two IDs were replaced with different IDs, so we used the updated IDs. Mouse gene symbols for the 842 Entrez IDs were pulled from org.Mm.eg.db. Human genes with Ago2 binding sites were taken from https://www.ncbi.nlm.nih.gov/pmc/articles/PMC4108341/bin/NIHMS553365-supplement-2.xlsx [28]. There were 7153 binding sites in 3416 unique Ensembl Gene IDs. Ensembl Gene IDs were converted to Entrez Gene IDs using the current Bioconductor org.Hs.eg.db database (version 3.4.0); 3173 Ensembl IDs had perfect 1:1 matches with Entrez IDs. There were 150 genes that had no matches based on Ensembl ID, but we were able to assign Entrez IDs for 109 of them using the Gene Symbol or RefSeq ID listed in https://www.ncbi.nlm.nih.gov/pmc/articles/PMC4108341/bin/NIHMS553365-supplement-2.xlsx. We found 93 genes that had more than one Entrez ID listed for the Ensembl ID; however, many of the Entrez IDs were for miRNA, and 57 genes matched to a single mRNA Entrez ID; an additional 33 genes were matched to a single Entrez ID using the Gene Symbol or RefSeq ID from https://www.ncbi.nlm.nih.gov/pmc/articles/PMC4108341/bin/NIHMS553365-supplement-2.xlsx. We were left with 3372 unique Ensembl genes, although a few of these were assigned the same Entrez ID, so there were 3335 unique Entrez Gene IDs that had Ago2 binding sites. Human gene symbols for these Entrez IDs were pulled from org.Hs.eg.db.

Mouse genes with Mov10 binding sites were taken from Additional file 11. There were a total of 930 binding sites, although 252 of these were in intergenic/intronic regions and were not assigned to a gene. An additional 2 sites were assigned to two different genes, and these were removed. The remaining sites were in 539 unique, current Entrez Gene IDs. Mouse gene symbols for the 539 Entrez IDs were pulled from org.Mm.eg.db.

While database IDs like Entrez Gene or Ensembl are much more stable over time than gene symbols, they are species-specific and hence cannot be used to easily map between species. Instead, both human and mouse use similar nomenclature systems for gene symbols, such that gene symbols from different databases *at the same point in time* should be comparable. The main difference is in capitalization, so symbols were matched by converting both to all capital letters. While only using genes with identical symbols between human and mouse will miss some true homologs, it simplifies the comparison by removing any many-to-one or many-to-many relationships that would be impossible to assess statistically. The two Bioconductor databases from the same release, org.Hs.eg.db_3.4.0 and org.Mm.eg.db_3.4.0, share 16,760 gene symbols in common. This gene set was used as the background to assess whether Fmrp, Ago2, and Mov10 tend to have binding sites in the same genes. After removing genes not in the background, we compared 821 Fmrp genes, 3130 Ago2 genes, and 502 Mov10 genes for overlaps (Fig. 5b). First, we compared the pairwise overlaps using a one-sided Fisher’s exact test, and all three sets were highly significant (Fmrp and Mov10, 77 genes in common, *p* = 2.15e-19; Fmrp and Ago2, 490 genes in common, *p* = 4.85e-159; Mov10 and Ago2, 193 in common, *p* = 5.57e-26). If we test the pairwise overlap between Fmrp and Mov10 using the full mouse background of 23,294 genes, their overlap of 80 genes is even more significant (*p* = 2.50e-27), indicating that the common background is actually more conservative. We also used a permutation approach to test whether the amounts of overlap in the Venn diagram were more than expected by chance by randomly selecting gene sets of 821, 3130, and 502 from the 16,760 background and counting the numbers of genes in common. We repeated this 50,000 times and used the resulting distributions of overlap values to empirically derive one-tailed *p* values for our four observed overlaps: (1) Fmrp and Mov10 only (30 genes), (2) Fmrp and Ago2 only (443 genes), (3) Mov10 and Ago2 only (146 genes), and (4) all three binding sites (47 genes). The Fmrp and Mov10 only overlap had a *p* value = 0.01116, which while significant is not nearly as significant as the other three overlap gene sets, which all had *p* = 0, meaning a larger overlap was not seen in 50,000 random samplings. R version 3.3.3 (R project for statistical Computing-RRID: SCR_001905), using a custom script to randomly pull out subsets of genes of the correct sizes and count overlaps, was used.

## Additional files

Additional file 1: Related to Fig. 1. Mov10 levels are elevated in Friend virus B-type (*FVB*) mice and are independent of sex. A) FVB brain (25 μg) at ages indicated, immunoblotted for Mov10 and eIF5α (loading control). Quantification of three independent experiments. Error bars represent SD, and *p* value indicates **p < 0.01 compared to adult. B) *Top panel*: 25 μg of P0 brains from 2 male and 2 female mice were immunoblotted for Mov10. 25 μg of adult brain lysate was used for comparison. *Bottom panel*: Genomic DNA was isolated from the P2 brain lysates of each mouse, and PCR was performed using SRY primers. Actin was used as a PCR control. (PDF 307 kb)

Additional file 2: Related to Fig. 1. Mov10 is nuclear in P0 cortex and hippocampal cultures compared to adult. A) DAB staining of P0 (*left*) and adult hippocampi (*right*) with Mov10. *Inset* shows the cellular localization of Mov10. Images obtained using the Hamamatsu NanoZoomer slide scanner. Scale bar = 250 μm. B) Immunofluorescence staining of Mov10 in P0 (*left*) and adult (*right*) cortex. Images obtained using the NanoZoomer slide scanning system. Scale bar = 100 μm. C) Immunofluorescence of endogenous Mov10 (*red*) at DIV1 (*top panel*), and DIV14 (*bottom panel*) in cultured primary hippocampal neurons. Nuclei were visualized with 4′,6-diamidino-2-phenylindole (*DAPI*). Scale bar = 20 μm for top panel and 10 μm for bottom panel. D) Immunohistochemistry of Mov10 in mouse testes sections from a WT male. Scale bar = 20 μm. E) Representative immunoblot from the nuclear fractionation of P2 brain (*n* = 3). 25 μg of purified nuclei preparations and cytoplasmic lysate was loaded, and the proportion of Mov10 in the supernatant (*S*) and pellet (*P*) was determined using immunoblotting. Transcription factor IID (*TFIID*) and histone were used as controls for fractionation. (PDF 13244 kb)

Additional file 3: Related to Table 1. Schematic of gene trap insertion into murine Mov10 gene to generate a knockout allele. Domain structure of Mov10 corresponding to exon sequence of murine Mov10 (NM_008619.2). Exons are shown as *black vertical lines*, and the gene trap vector is shown as inserting (*red arrow*) 3′ of start (ATG) in that exon. The resulting targeted allele is shown at the *bottom*. Gene trapping strategy is described in [69]. C57BL/6 embryonic stem cell (Clone IST13267G7sE6, RRID:IMSR_TIGM:IST13267G7) from the Texas A&M Institute for Genomic Medicine (*TIGM*) was used to generate the Mov10 heterozygote. (PDF 129 kb)

Additional file 4: Brain RIP-seq-repeat element identities. (XLSX 454 kb)

Additional file 5: Brain RIP-seq-mRNA identities. (XLSX 346 kb)

Additional file 6: Primary data for Fig. 2. (PDF 1809 kb)

Additional file 7: Brain iCLIP mRNA identities. (XLSX 71 kb)

Additional file 8: Related to Fig. 4. Mov10 binds mRNAs involved in actin cytoskeleton by RNA-seq. A) Screen shot from the sequencing of Mov10 exon2 in Mov10 KO N2a clone. *Top panel* is from WT Neuro2a. *Bottom panel* shows the mutant clone with the insertion generated by CRISPR-Cas9-mediated gene targeting. The mutation is boxed out. B) Gene Ontology (*GO*) analysis for Cellular Compartments from undifferentiated and differentiated WT Neuro2a. (See Additional file 9). (PDF 438 kb)

Additional file 9: RNA-seq of N2a WT undifferentiated versus WT differentiated N2a. (XLSX 5557 kb)

Additional file 10: Five hundred thirteen Mov10-dependent genes in N2a. (XLSX 36 kb)

Additional file 11: Mouse brain Mov10 clip targets. (XLSX 219 kb)

Additional file 12: Forty-seven brain Mov10/Fmrp/Ago2 cobound mRNAs. (XLSX 31 kb)

Additional file 13: Related to Fig. 7. Behavior testing of Mov10 heterozygotes. A) Rotarod testing was performed on both WT and Mov10 heterozygous littermates (*HET*) of both sexes (*n* = 11). No significant difference was found between sexes (WT, *p* = 0.44, Mov10 HET, *p* = 0.81; Student’s *t* test, two-tailed). Latency to fall (milliseconds) was calculated by averaging four trials per animal over 3 days. Error bars represent SEM. B) WT and Mov10 HETs (*n* = 10) of both sexes were used in the elevated plus maze; the percent time spent in the open and closed arms is plotted. Error bars represent SEM. C) Trace fear conditioning memory test: the level of freezing (percentage) in a new context with tone was assessed for WT and Mov10 HETs (*n* = 11). Both sexes were tested and no significant difference was found (WT, *p* = 0.33, Mov10 HET, *p* = 0.34; Student’s *t* test, two-tailed). Error bars represent SEM. D) Context fear memory test: the level of freezing (percentage) was measured on re-exposure to training context and is plotted for both WT and Mov10 HETs (*n* = 11). Both sexes were tested, and no significant difference was found (WT, *p* = 0.97, Mov10 HET, *p* = 0.38; Student’s *t* test, two-tailed). Error bars represent SEM. E) Percent novelty preference was calculated from interaction times {100× (time spent with novel object/time spent with both objects} and is plotted for WT (*n* = 10) and Mov10 HET (*n* = 12) males in the novel object recognition test. Error bars represent SEM. Student’s *t* test, one-tailed. (PDF 235 kb)

Additional file 14: Primers and plasmids. (PDF 50 kb)

## Abbreviations

Ago2: Argonaute 2
bp: Base pair
GO: Gene Ontology
iCLIP: Individual nucleotide cross-linking immunoprecipitation
IP: Immunoprecipitation
KO: Knockout
L1: Long interspersed nuclear elements 1
miRNA: MicroRNA
N2a: Neuro2A cells
RIP: RNA immunoprecipitation
RT: Reverse transcriptase, reverse transcription
SF1: Superfamily 1
WT: Wild-type

## References

[1] Gregersen LH (2014). MOV10 is a 5′ to 3′ RNA helicase contributing to UPF1 mRNA target degradation by translocation along 3′ UTRs. Mol Cell.

[2] Kenny PJ (2014). MOV10 and FMRP regulate AGO2 association with microRNA recognition elements. Cell Rep.

[3] Meister G (2005). Identification of novel argonaute-associated proteins. Curr Biol.

[4] Burdick R (2010). P body-associated protein Mov10 inhibits HIV-1 replication at multiple stages. J Virol.

[5] Goodier JL, Cheung LE, Kazazian HH (2012). MOV10 RNA helicase is a potent inhibitor of retrotransposition in cells. PLoS Genet.

[6] Abdelhaleem M (2010). Helicases: an overview. Methods in molecular biology.

[7] Bicker S (2013). The DEAH-box helicase DHX36 mediates dendritic localization of the neuronal precursor-microRNA-134. Genes Dev.

[8] Nicklas S (2015). The RNA helicase DDX6 regulates cell-fate specification in neural stem cells via miRNAs. Nucleic Acids Res.

[9] Li X (2013). The MOV10 helicase inhibits LINE-1 mobility. J Biol Chem.

[10] Coufal NG (2009). L1 retrotransposition in human neural progenitor cells. Nature.

[11] Banerjee S, Neveu P, Kosik KS (2009). A coordinated local translational control point at the synapse involving relief from silencing and MOV10 degradation. Neuron.

[12] Adams D (2013). Bloomsbury report on mouse embryo phenotyping: recommendations from the IMPC workshop on embryonic lethal screening. Dis Model Mech.

[13] Li Y (2013). RIPSeeker: a statistical package for identifying protein-associated transcripts from RIP-seq experiments. Nucleic Acids Res.

[14] DeBerardinis RJ (1998). Rapid amplification of a retrotransposon subfamily is evolving the mouse genome. Nat Genet.

[15] Heras SR (2013). The Microprocessor controls the activity of mammalian retrotransposons. Nat Struct Mol Biol.

[16] Muotri AR (2010). L1 retrotransposition in neurons is modulated by MeCP2. Nature.

[17] Najmudin S (2000). Crystal structures of an N-terminal fragment from Moloney murine leukemia virus reverse transcriptase complexed with nucleic acid: functional implications for template-primer binding to the fingers domain. J Mol Biol.

[18] Dlakic M, Mushegian A (2011). Prp8, the pivotal protein of the spliceosomal catalytic center, evolved from a retroelement-encoded reverse transcriptase. RNA.

[19] Iwatani Y (2007). Deaminase-independent inhibition of HIV-1 reverse transcription by APOBEC3G. Nucleic Acids Res.

[20] Wang X (2012). The cellular antiviral protein APOBEC3G interacts with HIV-1 reverse transcriptase and inhibits its function during viral replication. J Virol.

[21] Kopera HC (2016). LEAP: L1 Element Amplification Protocol. Methods Mol Biol.

[22] Moldovan JB (2015). Identification of cellular host factors that associate with LINE1-ORF1p and the effect of the zinc finger antiviral rotein ZAP on LINE-1 retrotransposition. Cellular and Molecular Biology. Thesis.

[23] Furtak V (2010). Perturbation of the P-body component Mov10 inhibits HIV-1 infectivity. PLoS ONE.

[24] Olmsted JB (1970). Isolation of microtubule protein from cultured mouse neuroblastoma cells. Proc Natl Acad Sci U S A.

[25] Kenny P, Ceman S (2016). RNA Secondary structure modulates FMRP’s bi-functional role in the microRNA pathway. Int J Mol Sci.

[26] Gomi F, Uchida Y (2012). MAP1B 1-126 interacts with tubulin isoforms and induces neurite outgrowth and neuronal death of cultured cortical neurons. Brain Res.

[27] Darnell JC (2011). FMRP stalls ribosomal translocation on mRNAs linked to synaptic function and autism. Cell.

[28] Boudreau RL (2014). Transcriptome-wide discovery of microRNA binding sites in human brain. Neuron.

[29] Wang B (2015). FMRP-mediated axonal delivery of miR-181d regulates axon elongation by locally targeting Map1b and Calm1. Cell Rep.

[30] Stark KL (2008). Altered brain microRNA biogenesis contributes to phenotypic deficits in a 22q11-deletion mouse model. Nat Genet.

[31] Hsu R (2012). Loss of microRNAs in pyramidal neurons leads to specific changes in inhibitory synaptic transmission in the prefrontal cortex. Mol Cell Neurosci.

[32] Ouchi Y (2013). Reduced adult hippocampal neurogenesis and working memory deficits in the Dgcr8-deficient mouse model of 22q11.2 deletion-associated schizophrenia can be rescued by IGF2. J Neurosci.

[33] Ferreira TA, Iacono LL, Gross CT (2010). Serotonin receptor 1A modulates actin dynamics and restricts dendritic growth in hippocampal neurons. Eur J Neurosci.

[34] Schneider CA, Rasband WS, Eliceiri KW (2012). NIH Image to ImageJ: 25 years of image analysis. Nat Methods.

[35] Kim IH (2015). Spine pruning drives antipsychotic-sensitive locomotion via circuit control of striatal dopamine. Nat Neurosci.

[36] Liu J (2004). Argonaute2 is the catalytic engine of mammalian RNAi. Science.

[37] Morita S (2007). One Argonaute family member, Eif2c2 (Ago2), is essential for development and appears not to be involved in DNA methylation. Genomics.

[38] Lykke-Andersen K (2008). Maternal Argonaute 2 is essential for early mouse development at the maternal-zygotic transition. Mol Biol Cell.

[39] Rice D, Barone S (2000). Critical periods of vulnerability for the developing nervous system: evidence from humans and animal models. Environ Health Perspect.

[40] Doucet AJ (2015). A 3′ poly(A) tract is required for LINE-1 retrotransposition. Mol Cell.

[41] Beck CR (2011). LINE-1 elements in structural variation and disease. Annu Rev Genomics Hum Genet.

[42] Howell R, Usdin K (1997). The ability to form intrastrand tetraplexes is an evolutionarily conserved feature of the 3′ end of L1 retrotransposons. Mol Biol Evol.

[43] Sahakyan AB (2017). G-quadruplex structures within the 3′ UTR of LINE-1 elements stimulate retrotransposition. Nat Struct Mol Biol.

[44] Nagase T (2000). Prediction of the coding sequences of unidentified human genes. XIX. The complete sequences of 100 new cDNA clones from brain which code for large proteins in vitro. DNA Res.

[45] Allen Developing Mouse Brain Atlas; 2008. http://developingmouse.brain-map.org/. Accessed 15 June 2017.

[46] Guffanti G (2014). Transposable elements and psychiatric disorders. Am J Med Genet B Neuropsychiatr Genet.

[47] McConnell MJ (2017). Intersection of diverse neuronal genomes and neuropsychiatric disease: The Brain Somatic Mosaicism Network. Science.

[48] Wegiel J (2010). The neuropathology of autism: defects of neurogenesis and neuronal migration, and dysplastic changes. Acta Neuropathol.

[49] McMurray CT (2000). Neurodegeneration: diseases of the cytoskeleton?. Cell Death Differ.

[50] Coe BP (2014). Refining analyses of copy number variation identifies specific genes associated with developmental delay. Nat Genet.

[51] Cooper GM (2011). A copy number variation morbidity map of developmental delay. Nat Genet.

[52] Kaminsky EB (2011). An evidence-based approach to establish the functional and clinical significance of copy number variants in intellectual and developmental disabilities. Genet Med.

[53] Miller DT (2010). Consensus statement: chromosomal microarray is a first-tier clinical diagnostic test for individuals with developmental disabilities or congenital anomalies. Am J Hum Genet.

[54] Kryukov K. FASTQ Splitter. 2014. http://kirill-kryukov.com/study/tools/fastq-splitter/. Accessed 15 June 2017.

[55] Konig J (2010). iCLIP reveals the function of hnRNP particles in splicing at individual nucleotide resolution. Nat Struct Mol Biol.

[56] Konig J (2011). iCLIP — transcriptome-wide mapping of protein-RNA interactions with individual nucleotide resolution. J Vis Exp.

[57] Huang DW, Sherman BT, Lempicki RA (2009). Systematic and integrative analysis of large gene lists using DAVID bioinformatics resources. Nat Protoc.

[58] Huang DW, Sherman BT, Lempicki RA (2009). Bioinformatics enrichment tools: paths toward the comprehensive functional analysis of large gene lists. Nucleic Acids Res.

[59] Dobin A (2013). STAR: ultrafast universal RNA-seq aligner. Bioinformatics.

[60] Trapnell C (2012). Differential gene and transcript expression analysis of RNA-seq experiments with TopHat and Cufflinks. Nat Protoc.

[61] Shannon P (2003). Cytoscape: a software environment for integrated models of biomolecular interaction networks. Genome Res.

[62] Merico D (2010). Enrichment Map: a network-based method for gene-set enrichment visualization and interpretation. PLoS One.

[63] Cong L (2013). Multiplex genome engineering using CRISPR/Cas systems. Science.

[64] Messaoudi-Aubert SE (2010). Role for the MOV10 RNA helicase in Polycomb-mediated repression of the INK4a tumor suppressor. Nat Struct Mol Biol.

[65] Beaudoin GM (2012). Culturing pyramidal neurons from the early postnatal mouse hippocampus and cortex. Nat Protoc.

[66] Faul F (2007). G*Power 3: a flexible statistical power analysis program for the social, behavioral, and biomedical sciences. Behav Res Methods.

[67] Leger M (2013). Object recognition test in mice. Nat Protoc.

[68] Konopka W (2010). MicroRNA loss enhances learning and memory in mice. J Neurosci.

[69] Hansen GM (2008). Large-scale gene trapping in C57BL/6 N mouse embryonic stem cells. Genome Res.

