# Supplementary figures and images for: Mov10 suppresses retroelements and regulates neuronal development and function in the developing brain
Source: BMC Biol. 2017 Jun 29;15:54. doi: 10.1186/s12915-017-0387-1 (PMC5492891; doi:10.1186/s12915-017-0387-1)

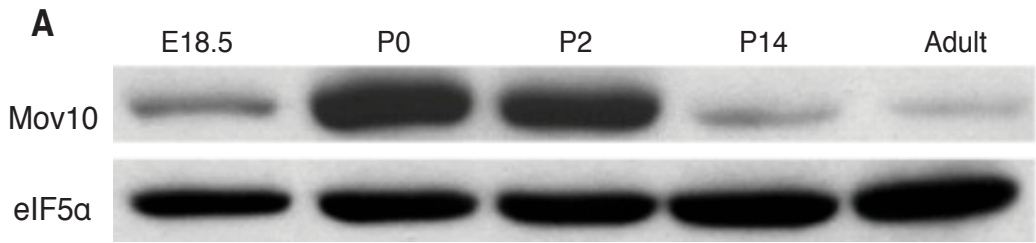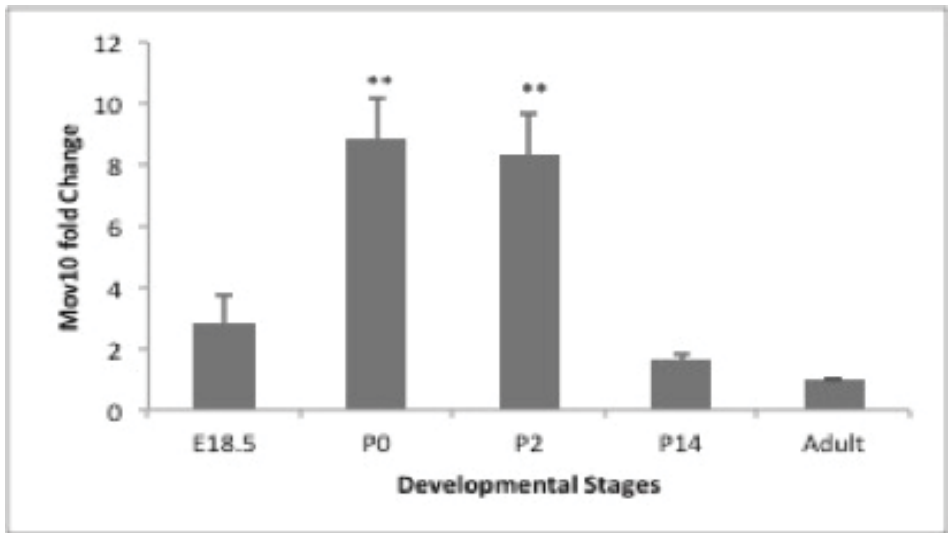

P0 Brains

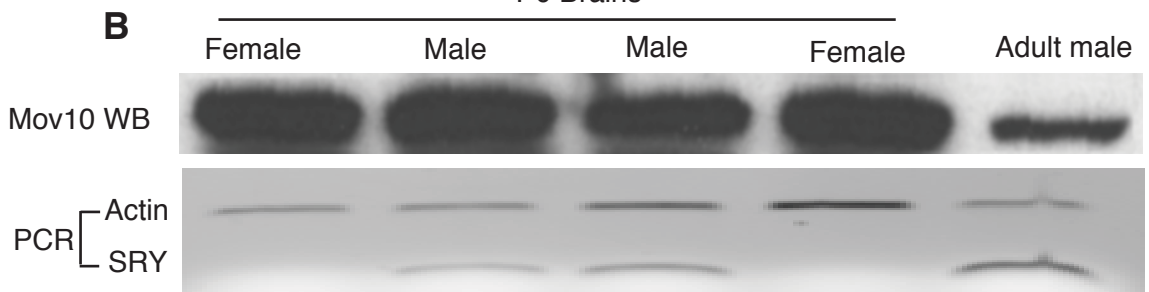

Supplement: Supplementary file 1 — Related to Fig. 1. Mov10 levels are elevated in Friend virus B-type (FVB) mice and are independent of sex. A) FVB brain (25 μg) at ages indicated, immunoblotted for Mov10 and eIF5α (loading control). Quantification of three independent experiments. Error bars represent SD, and p value indicates **p < 0.01 compared to adult. B) Top panel: 25 μg of P0 brains from 2 male and 2 female mice were immunoblotted for Mov10. 25 μg of adult brain lysate was used for comparison. Bottom panel: Genomic DNA was isolated from the P2 brain lysates of each mouse, and PCR was performed using SRY primers. Actin was used as a PCR control. (PDF 307 kb) [file 12915_2017_387_MOESM1_ESM.pdf]

**A**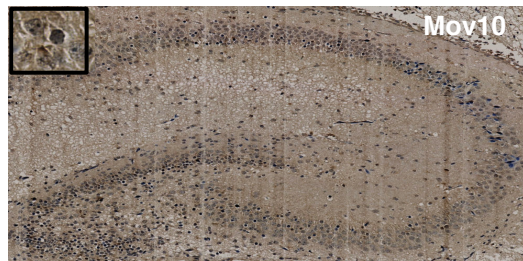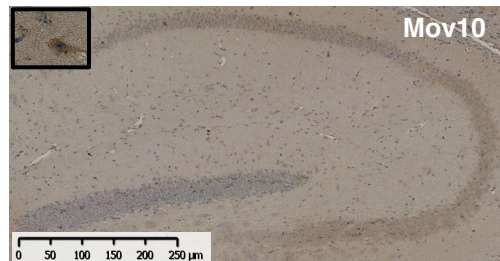**B**

P0 HC

Adult HC

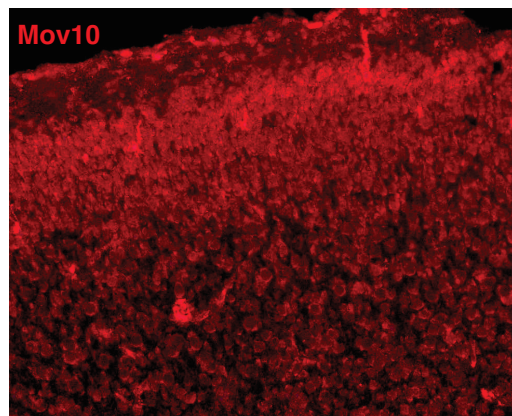

P0 cortex

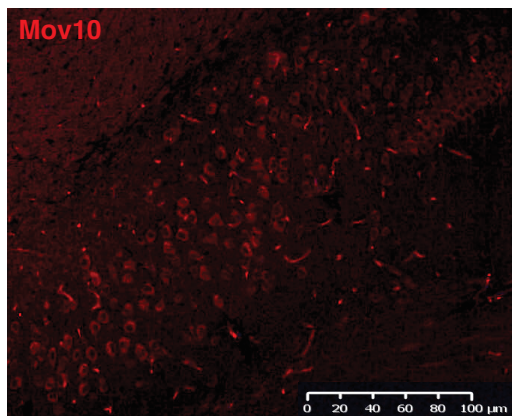

Adult cortex

**C**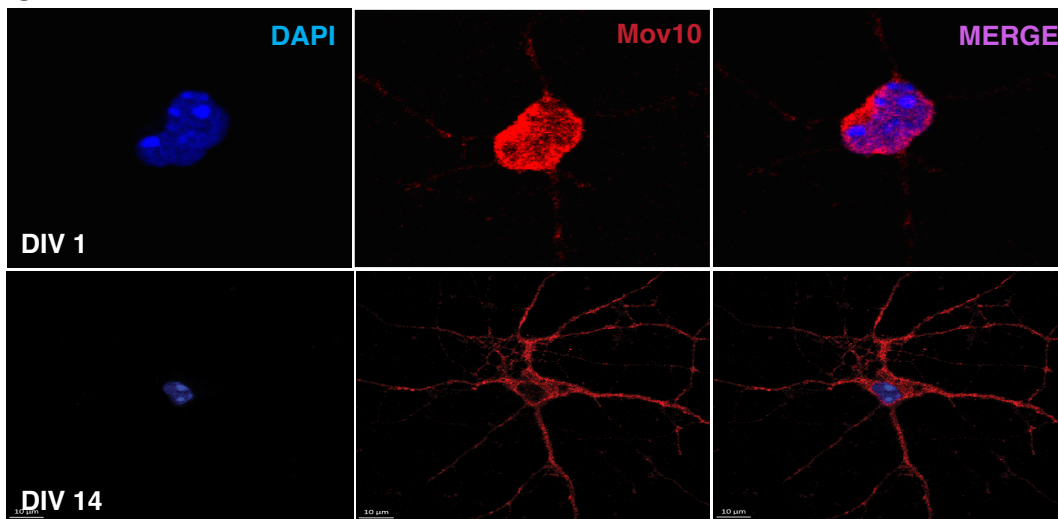**D**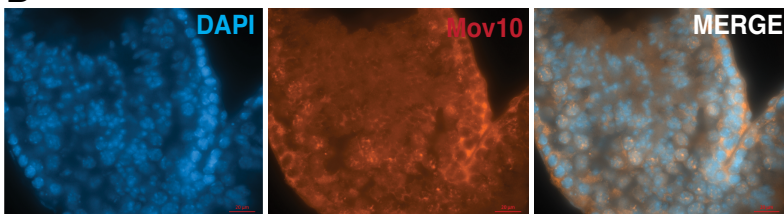**E**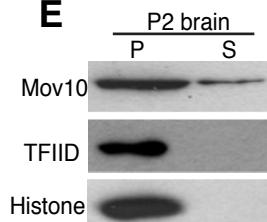

Supplement: Supplementary file 2 — Related to Fig. 1. Mov10 is nuclear in P0 cortex and hippocampal cultures compared to adult. A) DAB staining of P0 (left) and adult hippocampi (right) with Mov10. Inset shows the cellular localization of Mov10. Images obtained using the Hamamatsu NanoZoomer slide scanner. Scale bar = 250 μm. B) Immunofluorescence staining of Mov10 in P0 (left) and adult (right) cortex. Images obtained using the NanoZoomer slide scanning system. Scale bar = 100 μm. C) Immunofluorescence of endogenous Mov10 (red) at DIV1 (top panel), and DIV14 (bottom panel) in cultured primary hippocampal neurons. Nuclei were visualized with 4′,6-diamidino-2-phenylindole (DAPI). Scale bar = 20 μm for top panel and 10 μm for bottom panel. D) Immunohistochemistry of Mov10 in mouse testes sections from a WT male. Scale bar = 20 μm. E) Representative immunoblot from the nuclear fractionation of P2 brain (n = 3). 25 μg of purified nuclei preparations and cytoplasmic lysate was loaded, and the proportion of Mov10 in the supernatant (S) and pellet (P) was determined using immunoblotting. Transcription factor IID (TFIID) and histone were used as controls for fractionation. (PDF 13244 kb) [file 12915_2017_387_MOESM2_ESM.pdf]

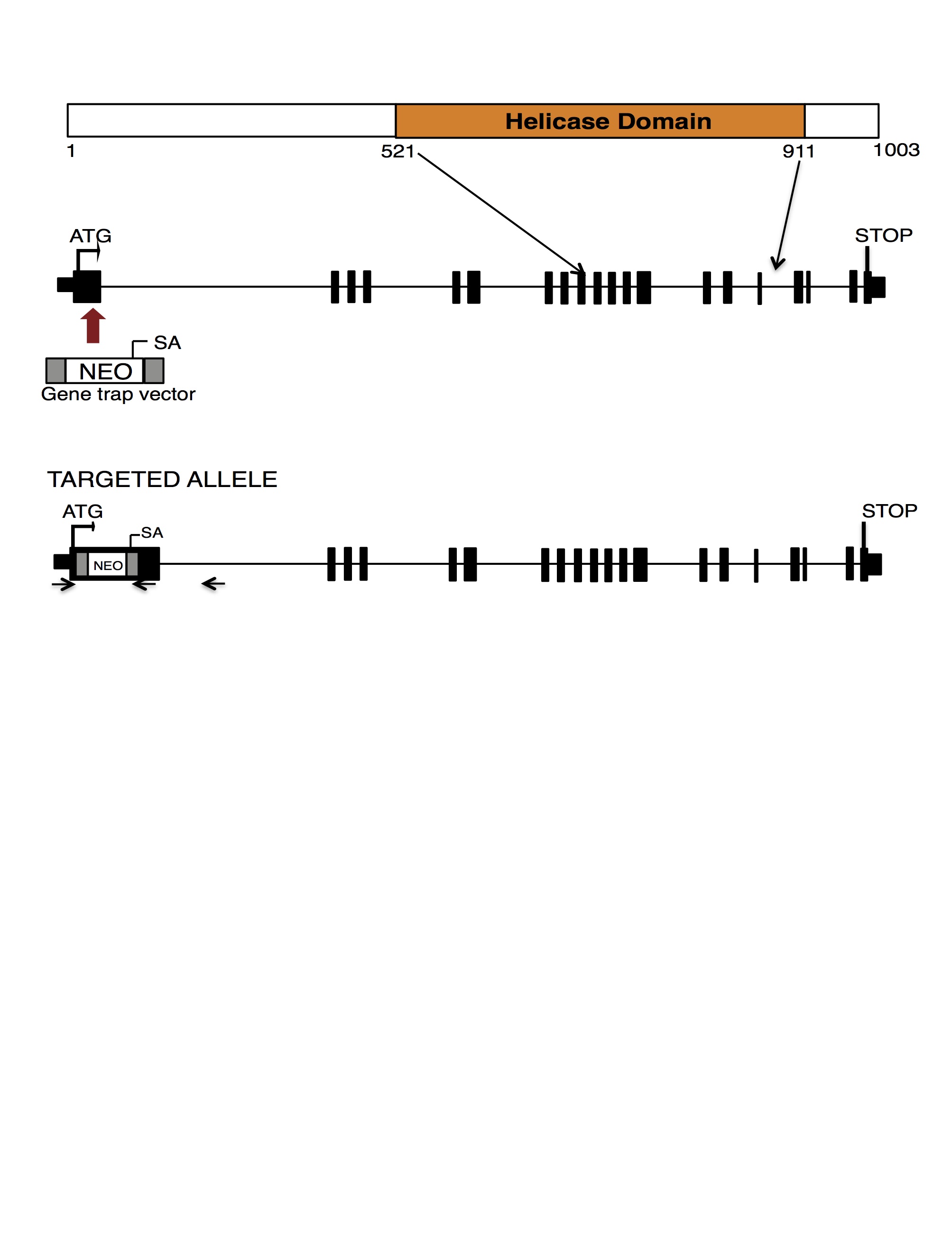

Supplement: Supplementary file 3 — Related to Table 1. Schematic of gene trap insertion into murine Mov10 gene to generate a knockout allele. Domain structure of Mov10 corresponding to exon sequence of murine Mov10 (NM_008619.2). Exons are shown as black vertical lines, and the gene trap vector is shown as inserting (red arrow) 3′ of start (ATG) in that exon. The resulting targeted allele is shown at the bottom. Gene trapping strategy is described in [69]. C57BL/6 embryonic stem cell (Clone IST13267G7sE6, RRID:IMSR_TIGM:IST13267G7) from the Texas A&M Institute for Genomic Medicine (TIGM) was used to generate the Mov10 heterozygote. (PDF 129 kb) [file 12915_2017_387_MOESM3_ESM.jpg]

**A**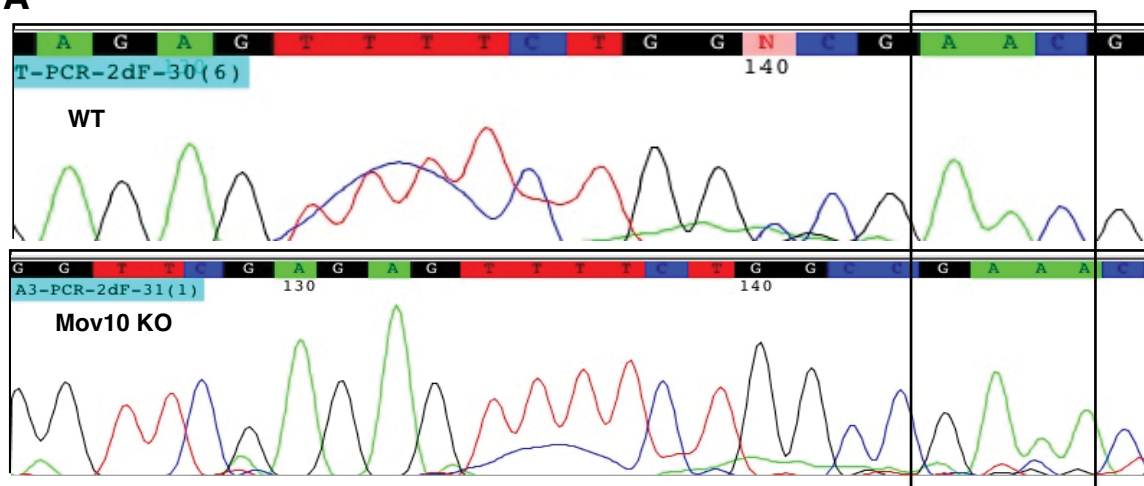**B**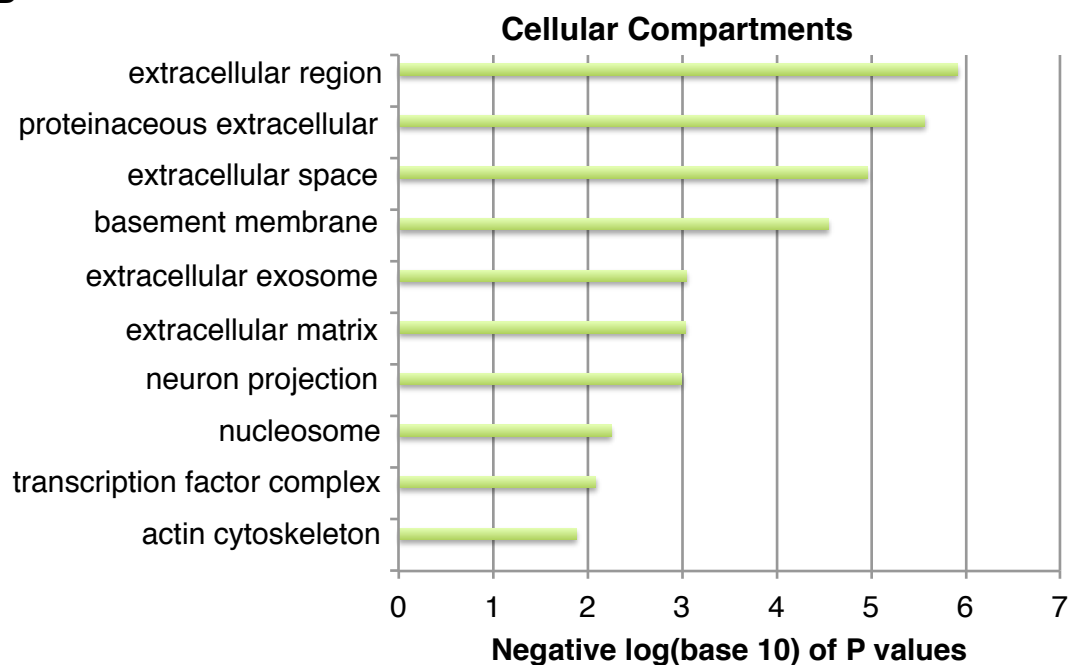

Supplement: Supplementary file 8 — Related to Fig. 4. Mov10 binds mRNAs involved in actin cytoskeleton by RNA-seq. A) Screen shot from the sequencing of Mov10 exon2 in Mov10 KO N2a clone. Top panel is from WT Neuro2a. Bottom panel shows the mutant clone with the insertion generated by CRISPR-Cas9-mediated gene targeting. The mutation is boxed out. B) Gene Ontology (GO) analysis for Cellular Compartments from undifferentiated and differentiated WT Neuro2a. (See Additional file 9). (PDF 438 kb) [file 12915_2017_387_MOESM8_ESM.pdf]

**A**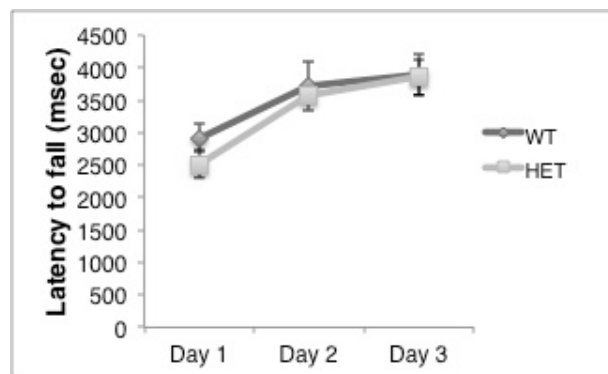**B**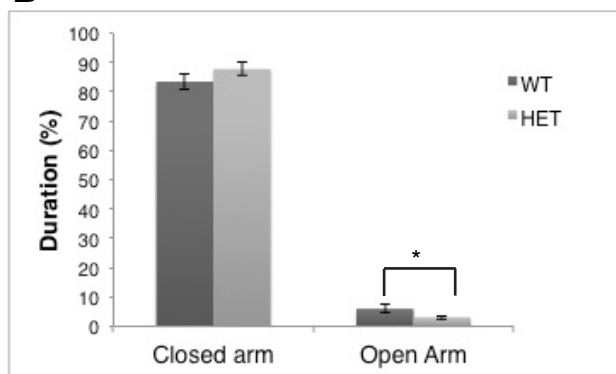**C**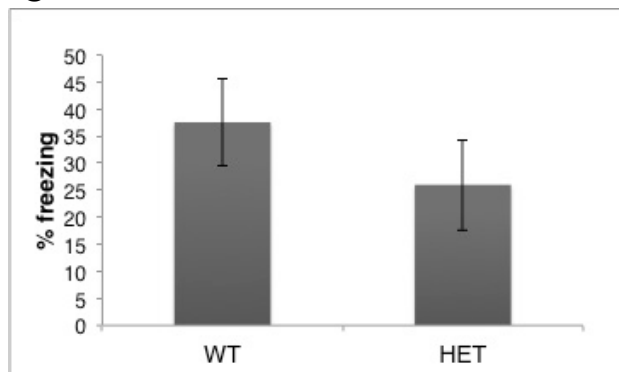**D**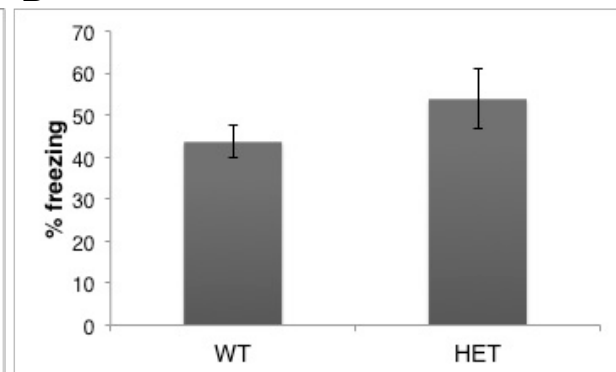**E**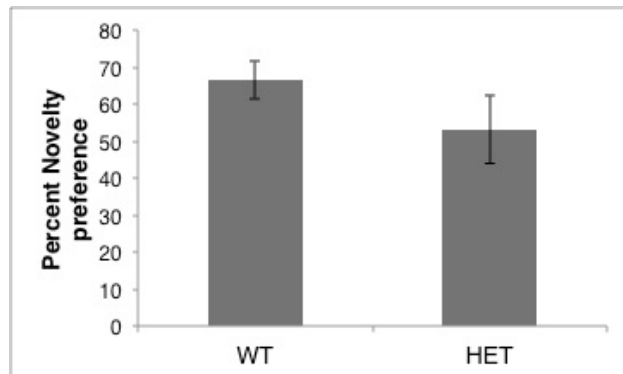

Supplement: Supplementary file 13 — Related to Fig. 7. Behavior testing of Mov10 heterozygotes. A) Rotarod testing was performed on both WT and Mov10 heterozygous littermates (HET) of both sexes (n = 11). No significant difference was found between sexes (WT, p = 0.44, Mov10 HET, p = 0.81; Student’s t test, two-tailed). Latency to fall (milliseconds) was calculated by averaging four trials per animal over 3 days. Error bars represent SEM. B) WT and Mov10 HETs (n = 10) of both sexes were used in the elevated plus maze; the percent time spent in the open and closed arms is plotted. Error bars represent SEM. C) Trace fear conditioning memory test: the level of freezing (percentage) in a new context with tone was assessed for WT and Mov10 HETs (n = 11). Both sexes were tested and no significant difference was found (WT, p = 0.33, Mov10 HET, p = 0.34; Student’s t test, two-tailed). Error bars represent SEM. D) Context fear memory test: the level of freezing (percentage) was measured on re-exposure to training context and is plotted for both WT and Mov10 HETs (n = 11). Both sexes were tested, and no significant difference was found (WT, p = 0.97, Mov10 HET, p = 0.38; Student’s t test, two-tailed). Error bars represent SEM. E) Percent novelty preference was calculated from interaction times {100× (time spent with novel object/time spent with both objects} and is plotted for WT (n = 10) and Mov10 HET (n = 12) males in the novel object recognition test. Error bars represent SEM. Student’s t test, one-tailed. (PDF 235 kb) [file 12915_2017_387_MOESM13_ESM.pdf]
