# Supplementary material for: Mov10 suppresses retroelements and regulates neuronal development and function in the developing brain
Source: BMC Biol. 2017 Jun 29;15:54. doi: 10.1186/s12915-017-0387-1 (PMC5492891; doi:10.1186/s12915-017-0387-1)
Supplement: Supplementary file 6 — Primary data for Fig. 2. (PDF 1809 kb) [file 12915_2017_387_MOESM6_ESM.pdf]

PRIMARY DATA

Related to Fig.2E – representative images. The samples were run on separate gels and all the gels used to generate the image in the paper are shown here. The lanes have been cropped and flipped to match the table above the figure.

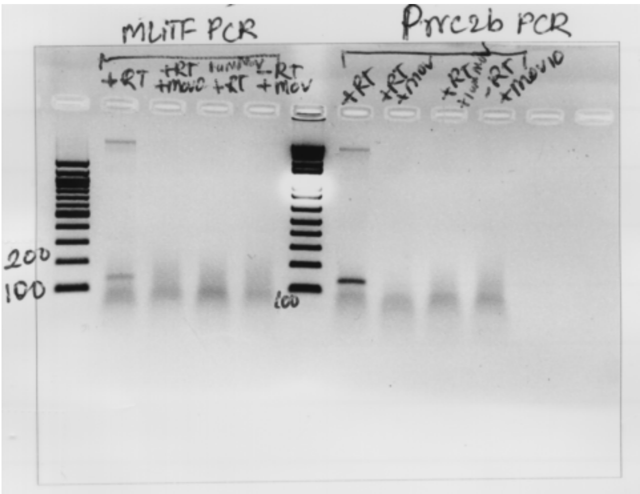

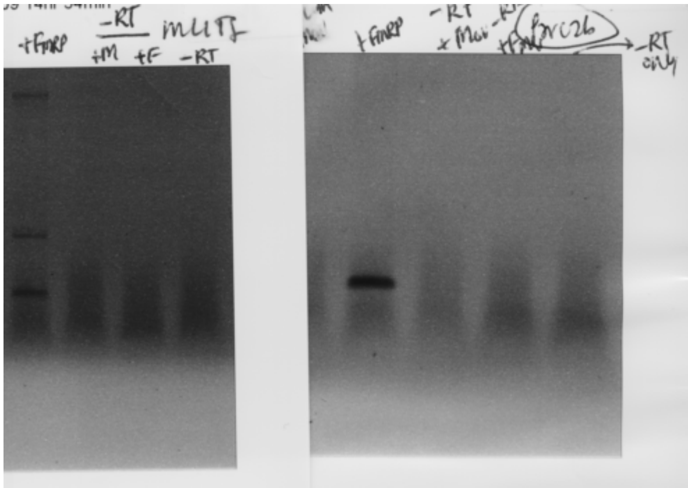

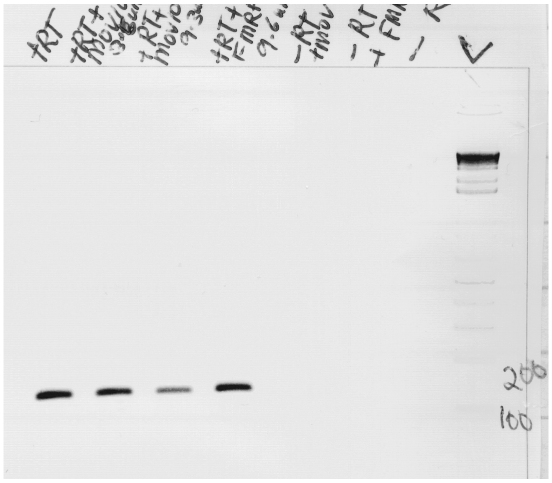

Related to Fig 2C, D. raw data (qRT) used to plot the graph for genomic LINE content

WT DNASE	HET DNAse
12.89000034	11.54246807
12.5947752	11.6178484
13.8006134	11.76618862
**13.09512965**	**11.64216836**
**0.628545915**	**0.113825818**

		deldel ct	power
WT-DNAse	13.1	1	
Het-Dnase	12	1.5	2.73769416037151

WT RnaseH-orf	Het RNASe H-orf
11.77509403	10.88058949
11.21201611	10.56461048
11.89022732	10.9873724
**11.62577915**	**10.81085745**
**0.362923976**	**0.219838183**

	del ct	deldel ct	power
WT RnaseH-orf	11.62577915		
Het RNASe H-orf	10.81085745	0.814921697	1.759202668

Related to Fig. 2F.

Data from biological replicates (first column) for RTase assay followed by qPCR . Please note- for mL1Tf, adding equimolar amounts of Mov10 generated an "undetermined" Ct value in qPCR in agreement with our observation. In order to do calculations for fold change; an arbitrary Ct of 35 was assigned.

**Prrc2b**		averages	del Ct	power	SD	p-value
+RT	17.03151321				0.560787759	
+RT	17.82458687	17.42805004	0	1		
+Rt/13mov	18.24653816				0.040993711	0.18546443
+Rt/13mov	18.1885643	18.21755123	-0.78950119	0.578544088		
equimolar	26.385669	26.461732	-	0.0019080	0.1075695	0.0019917

	71	86	9.033682823	53	47	33
equimolar	26.53779602					

**Gapdh**						
+RT	14.9251194	averages	del Ct	power		p-value
+RT	15.37994766	15.15253353	0	1	0.321612149	
+Rt/13mov	15.69124126					
+Rt/13mov	15.7839756	15.73760843	-0.585074902	0.666614728	0.065573078	0.127867224
equimolar	16.87296677					
equimolar	16.98036385	16.92666531	-1.774131775	0.29237021	0.075941203	0.016908373

**mL1Tf**		averages	del ct	power		p-value
+RT	26.16036415					
+RT	26.21929359	26.18982887	0	1	0.041669409	
+RT+1/3MOV	30.63841057					
+RT+1/3MOV	30.6096096	30.62401009	-4.434181213	0.046257105	0.020365357	5.46973E-05
equimolarRT	35					
equimolarRT	35	35	-8.810171127	0.00222779	0	1.11848E-05

	RT	1:0.3 uM	1:1uM
**Prrc2b**	1	0.578544088	0.001908053
**Gapdh**	1	0.666614728	0.29237021
**mL1Tf**	1	0.046257105	0.00222779
