# Supplementary material for: Mov10 suppresses retroelements and regulates neuronal development and function in the developing brain
Source: BMC Biol. 2017 Jun 29;15:54. doi: 10.1186/s12915-017-0387-1 (PMC5492891; doi:10.1186/s12915-017-0387-1)
Supplement: Supplementary file 14 — Primers and plasmids. (PDF 50 kb) [file 12915_2017_387_MOESM14_ESM.pdf]

LIST OF PRIMERS

Name of Primer	Sequence (5'-3')
ORF2-GDqRT-F	CTGGCGAGGATGTGGAGAA
ORF2-GDqRT-R	CCTGCAATCCCACCAACAAT
5S-Fwd	ACGGCCATACCACCCTGAA
5S-Rev	GGTCTCCCATCCAAGTACTAACCA
PRRC2B-F2	GGGTTTGCAGAAATCTGTCTCCAATT
PRRC2B-R2	ACCTTCGTGTCCGACTGGC
qRT-mL1TF-FWD	GAAATTAGTCTGAACAGGTGAGAGG
qRT-mL1TF-REV	TCCTCTGGTCCGGAAGGT
V76 F	CTTGCAAAATGGCGTTACTTAAGC
VP76 R	CCAATAAACCCTCTTGCAGTTGC
mMOV10-F	GCGATGCCTAGCAAGTTCAG
mMOV10-R-203	GCCAGATTTGCGATCTTCAT
mMOV10-R-250	CCCAGCGGTCTAGTTTGAAG
Sg2d-guide-seqFwd	CCCAGACCAGCCAACTCTAA
sg2d-guide-seq-Rev	GTGGTTGTAAATCGTCCGCA
SRY Fwd	TTGTCTAGAGAGCATGGAGGGCCATGTCAA
SRY Rev	CCACTCCTCTGTGACACTTTAGCCCTCCGA
Actin-geno Fwd	GATGTGCTCCAGGCTAAAGTT
Actin-geno Rev	AGAAACGGAATGTTGTGGAGT

LIST OF PLASMIDS

Plasmid name	Source
pX459	Addgene
Myc-mMOV10	Kenny et.al, Cell Reports, 2014
